# Comparative Efficacy of Chinese Herbal Injections for Treating Severe Pneumonia: A Systematic Review and Bayesian Network Meta-Analysis of Randomized Controlled Trials

**DOI:** 10.3389/fphar.2021.743486

**Published:** 2022-01-10

**Authors:** Liqing Niu, Lu Xiao, Xuemin Zhang, Xuezheng Liu, Xinqiao Liu, Xianglong Huang, Mingzhu Zhang

**Affiliations:** ^1^ Department of Emergency, First Teaching Hospital of Tianjin University of Traditional Chinese Medicine, Tianjin, China; ^2^ National Clinical Research Center for Chinese Medicine Acupuncture and Moxibustion, Tianjin, China; ^3^ State Key Laboratory of Multi-fractions Traditional Chinese Medicine, Tianjin University of Traditional Chinese Medicine, Tianjin, China

**Keywords:** network meta-analysis, severe pneumonia, Chinese herbal injections, combination therapy, antibiotic

## Abstract

**Background:** Severe pneumonia (SP) has a high mortality rate and is responsible for significant healthcare costs. Chinese herbal injections (CHIs) have been widely used in China as a novel and promising treatment option for SP. Therefore, this study assessed and ranked the effectiveness of CHIs to provide more sights for the selection of SP treatment.

**Method:** Seven databases were searched from their inception up to April 1, 2021. The methodological quality of included study was evaluated by the Cochrane risk-of-bias tool. Then, a Bayesian network meta-analysis (NMA) was performed by OpenBUGS 3.2.3 and STATA 14.0 software. The surface under the cumulative ranking curve (SUCRA) probability values were applied to rank the examined treatments. A clustering analysis was utilized to compare the effect of CHIs between two different outcomes.

**Results:** A total of 64 eligible randomized controlled trials (RCTs) involving 5,904 participants were identified for this analysis. Six CHIs including Xuebijing injection (XBJ), Tanreqing injection (TRQ), Reduning injection (RDN), Xiyanping injection (XYP), Shenfu injection (SF), and Shenmai injection (SM) were included. The results of the NMA showed that XBJ [odds ratio (OR) = 0.24, 95% credible interval (CI): 0.19, 0.30], TRQ (OR = 0.22, 95% CI: 0.12, 0.37), RDN (OR = 0.29, 95% CI: 0.04, 0.94), and SM (OR = 0.27, 95% CI: 0.08, 0.63) combined with conventional Western medicine (WM) improved the clinical effective rate more significantly than WM alone. Based on SUCRA values, TRQ + WM (SUCRA: 66.4%) ranked the highest in improving the clinical effective rate, second in four different outcomes, and third in only one. According to the cluster analysis, TRQ + WM exerted a positive effect on improving the efficacy of SP. As for safety, less than 30% (18 RCTs) of the included studies reported adverse drug reactions/adverse drug events (ADRs/ADEs), including 14 RCTs of XBJ, 3 RCTs of TRQ, and 1 RCT of RDN.

**Conclusion:** In conclusion, the study found that the CHIs as co-adjuvant therapy could be beneficial for patients with SP. TRQ + WM showed an outstanding improvement in patients with SP considering both the clinical effective rate and other outcomes.

**Systematic Review Registration:** [https://www.crd.york.ac.uk/prospero/], identifier [CRD42021244587].

## Introduction

Pneumonia is a persistent and pervasive burden of disease. A 2016 analysis of mortality trends reveals that pneumonia continues to cause more deaths in the USA than any other infectious disease, with no improvement at all during the preceding 34-year period analyzed (Hansen et al., 2016). Though not fatal, pneumonia can be severe. A fifth of the patients hospitalized for pneumonia need to be admitted to intensive care units (ICU), and a third of those require mechanical ventilation, while 21% of the patients from the community needed admission to the ICU, and 26% of them needed mechanical ventilation (Jain et al., 2015). Severe pneumonia (SP) remains a major cause of mortality and has a high mortality rate of up to 30%–50% (Restrepo et al., 2010; Prina et al., 2015; Lanks et al., 2019). The largest challenge in the next few years will be to diminish this high and unacceptable mortality rate. In addition, due to combined antibiotics, long mechanical ventilation, and hospitalization time, SP is responsible for significant healthcare costs (Welte, 2016).

Currently, therapies for SP mainly depend on antibiotics, mechanical ventilation, and corticosteroid (Metlay et al., 2019). Antibiotic therapy is the backbone of the management of SP. It must be started on an empiric basis and is often a combined therapy, since the causative agent is not identified in a considerable proportion of patients and the delay in the administration of adequate antimicrobials is clearly associated with mortality in patients (Garnacho-Montero et al., 2010). However, adequate initial antibiotics may elevate the risk of antibiotic resistance and the mortality rate (Musher and Thorner, 2014; Aliberti et al., 2019). The severity of pneumonia is determined by interacting processes of immune resistance and tissue resilience such as anti-inflammatory response (Mizgerd, 2017). Corticosteroids could inhibit the expression and action of many cytokines involved in the inflammatory response associated with pneumonia (Torres et al., 2015). However, the use of corticosteroids in clinical practice with SP remains controversial because of the presence of adverse effects (Bi et al., 2016; Wan et al., 2016). Therefore, there is an urgent need for combined and adjunctive therapeutic options to improve outcomes.

In recent years, Chinese herbal injections (CHIs) as adjuvant treatments for SP were widely applied in China (Qi et al., 2011; Lv et al., 2017; Song et al., 2019). Even in the treatment of COVID-19, CHIs displayed more superiority (Guo et al., 2020; Shi et al., 2021). Through clinical medication experience and searching in electronic databases previously, we found that six CHIs including Xuebijing injection (XBJ), Tanreqing injection (TRQ), Reduning injection (RDN), Xiyanping injection (XYP), Shenfu injection (SF), and Shenmai injection (SM) have been widely used in SP because of their remarkable effects. Their efficacy has been evidenced with systematic reviews (Huang et al., 2019; Jin et al., 2020; Zhou et al., 2021). CHIs combined with conventional Western medicine (WM) can greatly improve clinical symptoms and interrupt the vicious cycle of inflammation onset by blocking the uncontrolled release of endogenic inflammatory mediators like IL-6, IL-8, and TNF-α (Diao et al., 2017; Lv et al., 2017; Guo et al., 2020; Luo et al., 2021). However, head-to-head clinical trials comparing the efficacy of the six CHIs are lacking up to now. Without direct evidence, it is difficult to identify the most effective one for patients with SP. As a new method of evidence-based medical statistical methods, the network meta-analysis (NMA) extends principles of a conventional meta-analysis to the evaluation of multiple treatments in a single analysis by combining the direct and indirect evidence (Higgins and Welton, 2015; Shim et al., 2017). Another major value of NMA is that it can rank each CHI according to its effectiveness, which is important for clinicians to make the best treatment choices. Therefore, this study aimed to assess the clinical efficacy and safety of different CHIs combined with WM and provide more evidence for rational selection of CHIs for SP using NMA.

## Materials and Methods

### Study Registration

This study had been prepared under the guidance of the Preferred Reporting Items for Systematic Review and Meta-Analysis (PRISMA) guidelines (in Attachment 1) (Page et al., 2021). The study was prospectively registered on the PROSPERO platform (https://www.crd.york.ac.uk/prospero/) with an assigned registration number CRD42021244587.

### Inclusion and Exclusion Criteria

Studies were considered eligible if they met the following criteria: 1) randomized controlled trial (RCT). 2) Adults aged 18 years or older. 3) All included patients were diagnosed with SP according to the “Adult Community-Acquired Pneumonia (CAP) Guidelines for Diagnosis and Treatment” issued by the American Thoracic Society/American Society of Infectious Diseases (AST/IDSA) in 2007 (Mandell et al., 2007) or the “Guidelines for the Diagnosis and Treatment of Community-Acquired Pneumonia in Chinese Adults” developed by the Respiratory Branch of Chinese Medical Association in 2016 (Qu and Cao, 2016). 4) All patients received treatment with WM such as anti-infectives, phlegm reduction medicines, mechanical ventilation, nutritional support, and so on. Based on this, the experimental group received one of the included CHIs, and the control group received another or only WM. The duration of treatment ranged from 7 to 14 days. 5) At least one of the following seven outcomes with clear definitions were evaluated: the clinical effective rate, ICU length of stay, the time of mechanical ventilation, the level of C-reactive protein (CRP), the level of procalcitonin (PCT), leukocyte (WBC), and adverse drug reactions (ADRs)/adverse drug events (ADEs). The clinical effective rate was calculated by the following formula: (number of cured patients + number of improved patients)/total number of patients × 100%. Patients were regarded as cured when their clinical symptoms and the objective indicators disappeared and the patients returned to normal. Patients were regarded as improved when their clinical symptoms and the objective indicators were alleviated. If the clinical symptoms and objective indicators were either unchanged or aggravated, the patients were identified as having an invalid effectiveness status.

Studies were excluded if any of the following criteria were met: 1) If they described data about only a specific population (patients with tumor, pulmonary fibrosis, tuberculosis, using immunosuppressant, secondary respiratory failure in other systems, etc.). 2) The full text was not available or only with abstracts. 3) Data were incorrect, incomplete, or unavailable.

### Data Sources and Search Strategy

A comprehensive literature search was performed using the electronic databases of PubMed, the Cochrane Library, Embase, Web of Science, China National Knowledge Infrastructure (CNKI), Wanfang Database, and the Chinese Scientific Journal database (VIP) from their inception up to April 1, 2021. The medical subject headings (MeSH) and free text words were used. Language restriction did not exist in this study. Furthermore, we manually searched the reference lists of all retrieved studies. Six different kinds of CHIs were included in this NMA: TRQ, XYP, RDN, XBJ, SF, and SM. Full details of the search strategy are shown in Attachment 2.

### Study Selection and Data Extraction

Two researchers (LQ Niu and L Xiao) independently screened the studies according to the inclusion criteria. After checking for duplicate studies, the researchers eliminated reviews and irrelevant studies by reading the titles and abstracts. Then, full texts were read to select studies that meet the pre-specified inclusion criteria. Inconsistencies were resolved by extensive discussion or the third researcher (XZ Liu). A data spreadsheet was developed with Microsoft Excel 2019 to collect relevant information. The information including eligible study characteristics (e.g., first author and year of publication), participant characteristics (e.g., gender, age, and sample), details of interventions (e.g., duration and frequency of drugs), outcome data, and factors to evaluate risk of bias were extracted and entered into the spreadsheet.

### Quality Assessment

The methodological quality of each included study was evaluated with Revised Cochrane risk-of-bias tool for randomized trials (RoB 2) (Sterne et al., 2019). The domains include the following: 1) randomization process, 2) deviations from intended interventions, 3) missing outcome data, 4) measurement of the outcome, and 5) selection of the reported result. There are some signaling questions required to be answered by “yes (Y),” “probably yes (PY),” “probably no (PN),” “no (N),” or “no information (NI)” for each domain. After that, the risk of bias is categorized into three levels: high risk, some concerns, and low risk. These domain-level judgements will inform an overall risk of bias judgment for the outcome. The quality assessments were performed by two independent reviewers (LQ Niu and L Xiao), and disagreements were resolved by consensus or a third opinion.

### Statistical Analysis

OpenBUGS 3.2.3 (Lunn et al., 2009) and STATA 14.0 software (Stata Corporation, College Station, TX, USA) were employed to compute calculations and prepare graphs. For binary outcomes, the combined results were calculated as odds ratios (ORs) with 95% credible intervals (CIs). For continuous outcomes, standardized mean differences (SMD) with 95% CIs were used. When the 95% CIs of ORs did not include one and the 95% CIs of the SMDs did not contain zero, the differences between the groups were considered statistically significant. If there were multi-arm trials, we split them into two-arm trials. Chi-squared test was employed to assess heterogeneity between different studies (Zheng et al., 2019). If with homogeneity (*p *≥ 0.1, *I* ^2^ ≤ 50%), a fixed-effect model was adopted; if with obvious heterogeneity (*p* < 0.1, *I* ^2^ > 50%), a random-effect model was applied. If closed loops existed, we employed the inconsistency factor (IF) to examine the consistency between direct and indirect evidence. If 95% CIs of IF values were truncated at zero, it indicated that the two sources are in agreement (Salanti et al., 2011). Due to non-closed loops in this NMA, the assumption of consistency between direct and indirect evidence was not utilized.

The Markov chain Monte Carlo method was performed by using the OpenBUGS software to carry out the NMA. In OpenBUGS software, the number of iterations was set to 300,000, and the first 100,000 iterations were used for the annealing algorithm to eliminate the impact of the initial value. The network graph was constructed using STATA software to show a comparative relationship between different interventions. Surface under the cumulative ranking curve (SUCRA) probability values were applied to rank the examined treatments, and the SUCRA values of 100% and 0% were assigned to the best and worst treatments, respectively (Salanti et al., 2011; Riley et al., 2017). Furthermore, a clustering analysis was utilized to compare the effect of CHIs between two different outcomes. After that, publication bias were reflected by funnel plots (Chaimani et al., 2013; Salanti et al., 2014).

## Results

### Literature Selection

**FIGURE 1:**
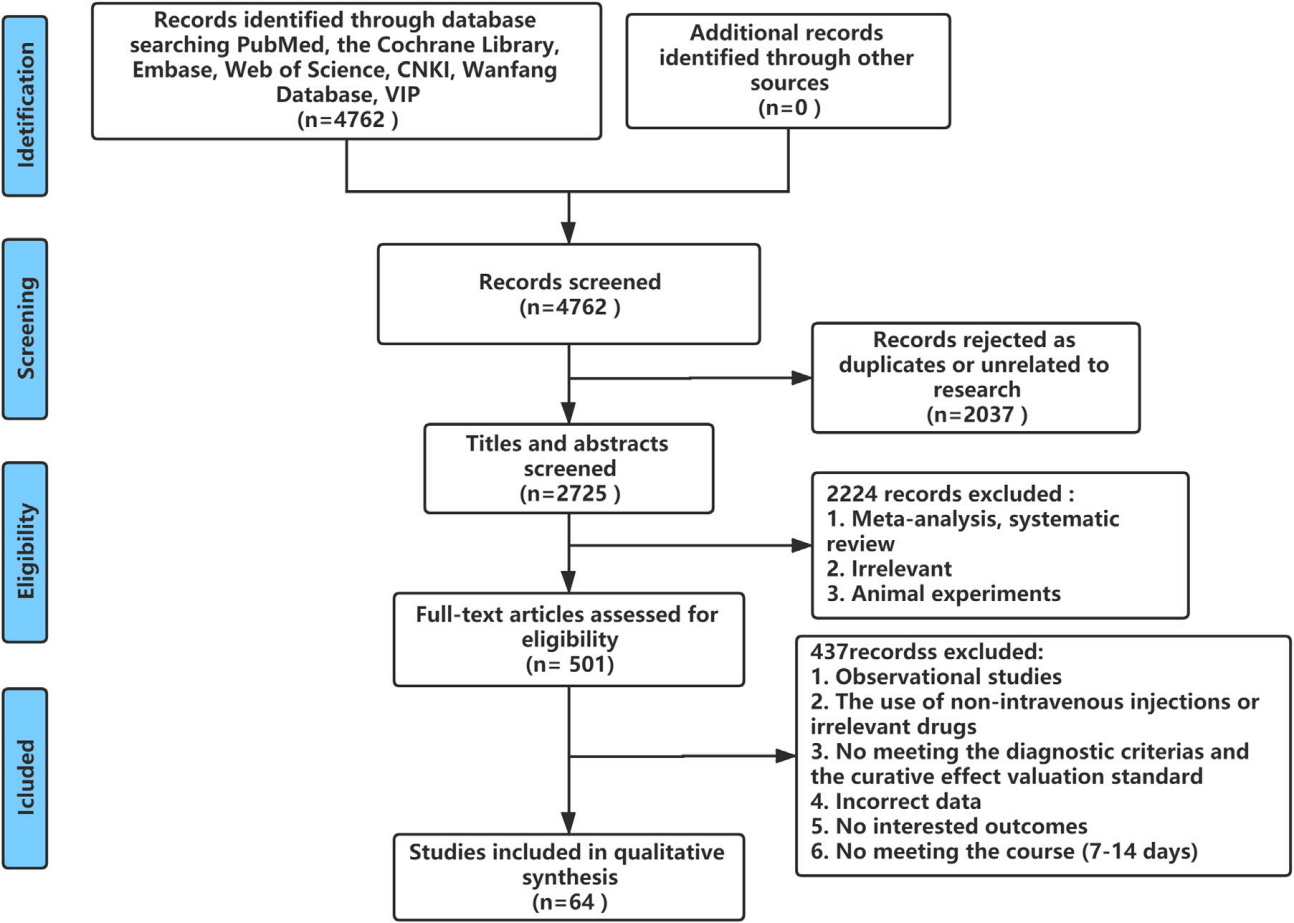
Flow diagram of study inclusion.

A total of 4,762 studies were identified from the search at first. After removing duplicates, 2,725 remained. By screening titles and abstracts, 2,224 studies were excluded because they were reviews, irrelevant studies, and animal experiments. Afterward, 501 relevant studies were reviewed for eligibility by full-text evaluations. Finally, 64 studies that met the inclusion criteria were included in our Bayesian NMA. Four hundred thirty-seven records were excluded for the following reasons: 1) observational studies (*n* = 33); 2) the use of non-intravenous injections or irrelevant drugs (*n* = 63); 3) the disease did not meet the diagnostic criteria or studies did not report the established outcomes (*n* = 327); 4) incomplete data (*n* = 6); and 5) duration of therapy was not satisfied (*n* = 8). The literature selection process is illustrated in Figure 1.

### Study Characteristics

**FIGURE 2:**
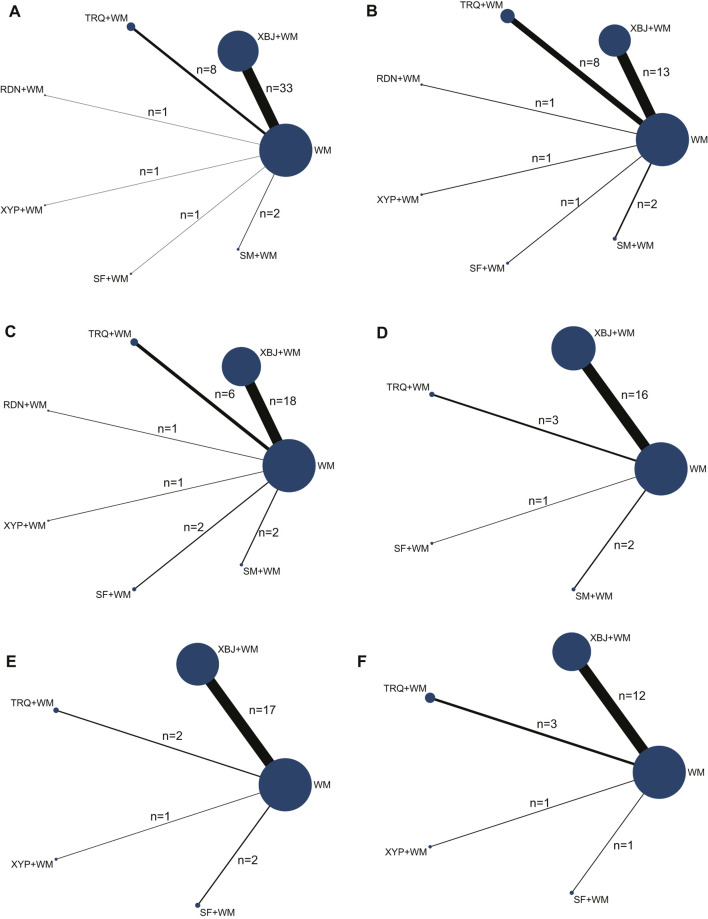
Network graph of the different outcomes. **(A)** Clinical effective rate; **(B)** the level of leukocyte (WBC); **(C)** the level of C-reactive protein (CRP); **(D)** the level of procalcitonin (PCT); **(E)** intensive care unit (ICU) length of stay; and **(F)** the time of mechanical ventilation.

The Bayesian NMA was performed using 64 RCTs with a total of 5,904 adult patients and their sample sizes varying from 24 to 675 participants. All RCTs were conducted in China and published between 2011 and 2021. Six CHIs were incorporated, including XBJ (*n* = 46), TRQ (*n* = 10), RDN (*n* = 1), XYP (*n* = 2), SF (*n* = 3), and SM (*n* = 2). The control groups have been treated with WM such as anti-infectives, apophlegmatisant, mechanical ventilation, vasopressor, and nutritional support. The anti-infectives mainly include fluoroquinolones, β-lactam, carbapenems, and linezolid as monotherapy or combination therapy. On the basis of the control group, the intervention of the experimental group was one of the included CHIs. The duration of treatment ranged from 7 to 14 days. The details of the study characteristics are depicted in Table 1, and the compared connections among each intervention for each outcome are displayed in Figure 2.

**TABLE 1:** Characteristics of the studies included in this meta-analysis.

Study ID	*N* (E/C)	Sex (M/F)	Age (years)	Therapy of experiment group	Therapy of control group	Course (day)	Outcomes
Qi et al. (2011)	40/40	48/32	–	Xuebijing 50 ml q12 h + WM	WM	14	123
Li (2017)	40/40	48/32	E: 65 ± 2.1; C: 70 ± 2.5	Xuebijing 50 ml q12 h + WM	WM	10	1
Zhou et al. (2017)	30/30	35/25	E: 68.7 ± 4.5; C: 69.4 ± 5.2	Xuebijing 50 ml q12 h + WM	WM	14	1
Yin (2019)	34/34	39/29	E: 46.62 ± 10.46; C: 46.59 ± 10.37	Xuebijing 50 ml q12 h + WM	WM	14	1
Shang and Zhang (2019)	102/102	110/94	E: 53.96 ± 3.68; C: 53.69 ± 3.47	Xuebijing 50 ml q12 h + WM	WM	14	1,237
Chen et al. (2019)	50/50	57/43	E: 53.45 ± 9.71; C: 53.78 ± 11.36	Xuebijing 50 ml q12 h + WM	WM	10	1,235
Xin (2020)	60/60	65/55	E: 51.43 ± 5.83; C: 51.55 ± 5.89	Xuebijing 50 ml q12 h + WM	WM	14	147
Xiao (2020)	48/48	51/45	E: 69.13 ± 5.24; C: 69.22 ± 4.57	Xuebijing 50 ml q12 h + WM	WM	14	1,345
Chen et al. (2019)	43/43	45/41	E: 69.78 ± 5.76; C: 68.23 ± 4.92	Xuebijing 50 ml q12 h + WM	WM	14	234
Meng and Xie (2018)	41/40	37/44	E: 48.34 ± 12.19; C: 48.04 ± 12.44	Xuebijing 50 ml q12 h + WM	WM	14	17
Wang (2019)	44/44	47/41	E: 48.82 ± 6.53; C: 49.37 ± 6.28	Xuebijing 50 ml q12 h + WM	WM	14	1
Zhou (2018)	42/42	44/40	E: 43.1 ± 1.5; C: 42.6 ± 1.4	Xuebijing 50 ml q12 h + WM	WM	14	157
Wang et al. (2020)	42/42	39/45	E: 47.04 ± 10.12; C: 46.91 ± 9.78	Xuebijing 50 ml q12 h + WM	WM	14	1
Wang et al. (2017)	30/30	32/28	E: 59.35 ± 6.28; C: 59.41 ± 6.30	Xuebijing 50 ml q12 h + WM	WM	14	1
Tian et al. (2019)	37/37	42/32	E: 50.23 ± 6.23; C: 49.89 ± 6.87	Xuebijing 50 ml q12 h + WM	WM	10	134
Zhang et al. (2014)	30/30	33/27	E: 51.2 ± 9.4; C: 49.8 ± 8.5	Xuebijing 50 ml q12 h + WM	WM	14	12,357
Xie et al. (2016)	50/50	55/45	E: 52.13 ± 2.10; C: 55.60 ± 2.10	Xuebijing 50 ml q12 h + WM	WM	14	123
Qiu (2021)	43/43	45/31	E: 68.65 ± 2.31; C: 68.47 ± 2.19	Xuebijing 50 ml q12 h + WM	WM	14	17
Xu (2017)	47/47	57/37	E: 54.8 ± 2.61; C: 54.3 ± 2.5	Xuebijing 50 ml q12 h + WM	WM	7–14	123
Ding and Lu (2020)	20/20	24/16	E: 51.46 ± 8.25; C: 51.62 ± 8.38	Xuebijing 50 ml q12 h + WM	WM	14	13
Sheng et al. (2019)	21/21	30/12	E: 43.57 ± 7.22; C: 44.33 ± 8.89	Xuebijing 50 ml q12 h + WM	WM	–	45
Wang (2019)	49/49	58/40	E: 54.77 ± 8.05; C: 53.26 ± 8.79	Xuebijing 50 ml q12 h + WM	WM	7–8	1,567
Wang (2018)	38/38	43/33	E: 58.4 ± 6.7; C: 57.6 ± 5.2	Xuebijing 50 ml q12 h + WM	WM	7	156
Wang (2019)	34/34	42/26	E: 75.2 ± 11.7; C: 75.6 ± 10.1	Xuebijing 50 ml q12 h + WM	WM	7	147
Wei and Huang (2020)	25/25	35/15	E: 65.8 ± 1.5; C: 65.9 ± 1.4	Xuebijing 50 ml q12 h + WM	WM	7	156
Wu and Ren (2016)	100/100	122/78	70 ± 3.4	Xuebijing 50 ml q12 h + WM	WM	7	1,247
Yang et al. (2020)	16/15	21/10	E: 70.5 ± 9.5; C: 69.0 ± 8.6	Xuebijing 50 ml q12 h + WM	WM	7	4
Yang (2020)	52/51	49/54	E: 53.39 ± 9.16; C: 52.45 ± 9.26	Xuebijing 50 ml q12 h + WM	WM	7	346
Chen et al. (2014)	40/40	–	–	Xuebijing 50 ml q12 h + WM	WM	7	456
Diao et al. (2017)	50/50	58/42	43.8 ± 18.1	Xuebijing 50 ml q12 h + WM	WM	7	23
Zheng et al. (2020)	51/52	53/50	E: 62.34 ± 13.79; C: 61.78 ± 13.42	Xuebijing 50 ml q12 h + WM	WM	7	1
Zhu and Liu (2014)	22/21	30/13	E: 68.3 ± 8.0; C: 64.6 ± 4.5	Xuebijing 50 ml q12 h + WM	WM	–	56
Zhuang (2016)	12/12	–	–	Xuebijing 50 ml q12 h + WM	WM	7	23
Zhao et al. (2019)	20/20	30/10	E: 42.37 ± 6.22; C: 43.43 ± 7.89	Xuebijing 50 ml q12 h + WM	WM	7	45
Pan et al. (2017)(1)	45/46	67/24	E: 69.7 ± 3.9; C: 69.2 ± 4.1	Xuebijing 50 ml q12 h + WM	WM	7	1,234,567
Pan (2017)(2)	47/46	64/29	E: 70.6 ± 4.0; C: 69.2 ± 4.1	Xuebijing 100 ml q12 h + WM	WM	7	1,234,567
Song et al. (2019)	334/341	458/217	E: 58.67 ± 13.58; C: 58.13 ± 14.24	Xuebijing 100 ml q12 h + WM	WM + placebo	7	347
Wu (2015)	65/61	73/53	74 ± 3	Xuebijing 100 ml q12 h + WM	WM	14	1
Song and Chai (2015)	41/40	42/39	–	Xuebijing 100 ml q12 h + WM	WM	10	56
Gao et al. (2014)	63/63	70/56	E: 50.2 ± 18.9; C: 51.0 ± 19.2	Xuebijing 100 ml q12 h + WM	WM	14	23,456
Ma (2016)	30/30	32/28	E: 49.3 ± 16.5; C: 49.7 ± 16.2	Xuebijing 100 ml q12 h + WM	WM	14	1,567
Niu et al. (2017)	48/48	53/43	E: 66 ± 8; C: 66 ± 8	Xuebijing 100 ml q12 h + WM	WM	14	1
Yuan and Zhao (2020)	46/46	45/47	E: 51.32 ± 2.18; C: 50.24 ± 3.15	Xuebijing 100 ml q12 h + WM	WM	10	34
Deng and Chen (2021)	75/75	85/65	E: 47.89 ± 11.35; C: 47.12 ± 11.21	Xuebijing 100 ml q12 h + WM	WM	7	1,567
Han et al. (2012)	31/31	42/20	57.5 ± 28.1	Xuebijing 100 ml q12 h + WM	WM	7	56
Kong (2015)	34/34	57/11	E: 48.90 ± 10.10; C: 50.60 ± 12.20	Xuebijing 100 ml q12 h + WM	WM	7	1,234
Zhang et al. (2020)	40/40	48/32	E: 52.14 ± 1.42; C: 51.86 ± 1.36	Xuebijing 100 ml q12 h + WM	WM	7	1
Cai (2020)	30/30	37/23	E: 67.12 ± 9.21; C: 68.75 ± 8.97	Tanreqing 10 ml qd + WM	WM	14	12,347
Lei et al. (2017)	23/21	34/10	E: 55.33 ± 1.96; C: 54.15 ± 2.78	Tanreqing 20 ml qd + WM	WM	14	235
Huang (2015)	50/50	53/47	E: 53.56 ± 5.47; C: 52.38 ± 5.21	Tanreqing 20 ml qd + WM	WM	14	127
Wu (2012)	30/30	41/19	78.62 ± 13.48	Tanreqing 20 ml qd + WM	WM	10	1
Wu and Ru (2011)	53/19	47/25	–	Tanreqing 20 ml qd + WM	WM	10–14	16
Zhang (2021)	25/25	27/23	E: 70.53 ± 1.04; C: 70.55 ± 1.01	Tanreqing 20 ml qd + WM	WM	14	12,347
Xi et al. (2016)	40/40	44/36	–	Tanreqing 20 ml qd + WM	WM	10	1,237
Li (2013)	72/36	56/52	78.35 ± 3.82	Tanreqing 40 ml qd + WM	WM	14	256
Sun et al. (2020)	36/36	40/32	E: 71.45 ± 8.60; C: 71.36 ± 8.52	Tanreqing 40 ml qd + WM	WM	14	123
Liu and Qin (2019)	46/46	44/48	E: 70.25 ± 3.33; C: 70.16 ± 3.28	Tanreqing 40 ml qd + WM	WM	14	12,346
Sun et al. (2012)	33/31	33/31	–	Reduning 20 ml qd + WM	WM	7–10	1,237
Yang et al. (2014)	34/34	32/36	E: 76.35 ± 7.21; C: 75.52 ± 6.47	Xiyanping 20 ml qd + WM	WM	7	1,256
Zhang and Wang (2015)	33/30	35/28	E: 74.82 ± 12.02; C: 73.82 ± 11.97	Xiyanping 375 mg qd + WM	WM	14	3
Lv et al. (2017)	45/44	51/38	E: 67.2 ± 15.0; C: 65.4 ± 6.7	Shenfu 50 ml q12 h + WM	WM	7	2,356
Lin et al. (2013)	33/16	29/20	E: 79.80 ± 12.60; C: 76.50 ± 13.20	Shenfu 120 ml q12 h + WM	WM	–	5
Xia and Xie (2017)	47/47	57/37	E: 72.37 ± 5.65; C: 71.84 ± 6.23	Shenfu 120 ml q12h + WM	WM	7	134
Fan et al. (2018)	30/30	35/25	E: 65.3 ± 6.7; C: 65.7 ± 7.1	Shenmai 100 ml qd + WM	WM	10	1,234
Yang and Zhang (2019)	38/38	39/37	E: 76.37 ± 6.69; C: 74.12 ± 6.85	Shenmai 100 mg Bid + WM	WM	7	1,234

Note: 1 clinical effective rate; 2 the level of WBC; 3 the level of CRP; 4 the level of PCT; 5 ICU length of stay; 6 the time of mechanical ventilation; 7 adverse drug reactions/adverse drug events (ADRs/ADEs).

### Quality Assessments of Studies

**FIGURE 3:**
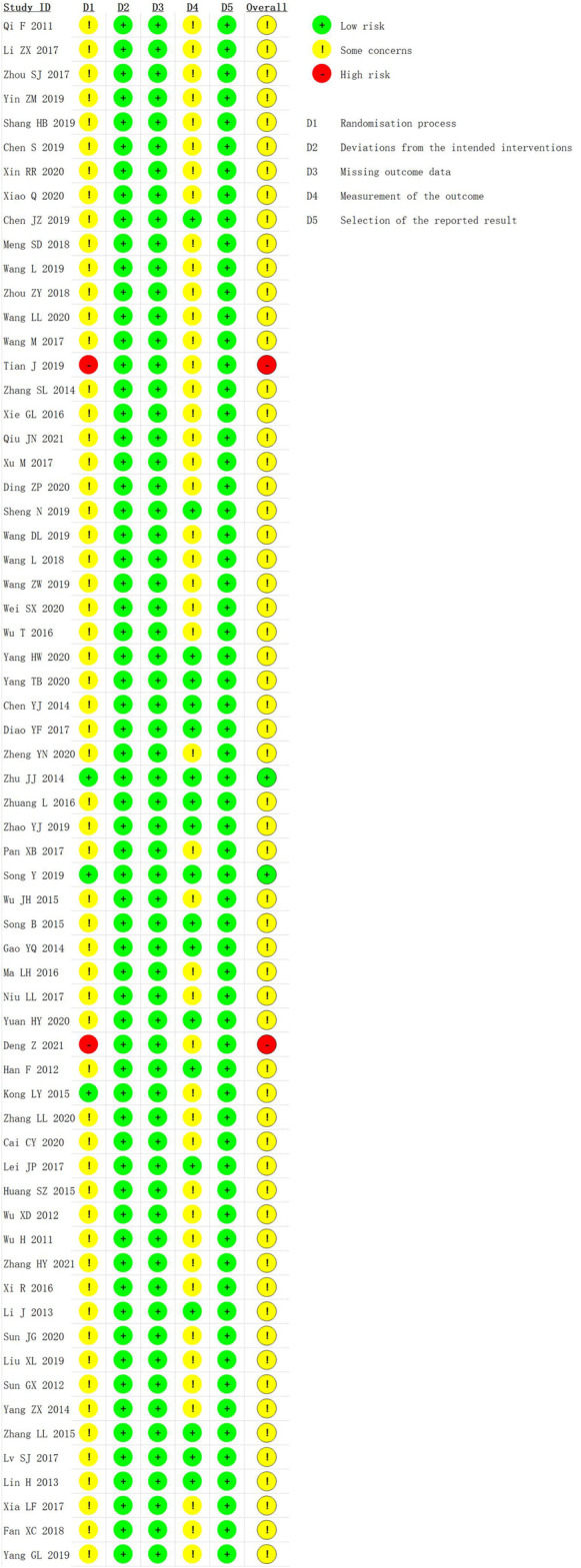
Risk-of-bias graph.

**FIGURE 4:**
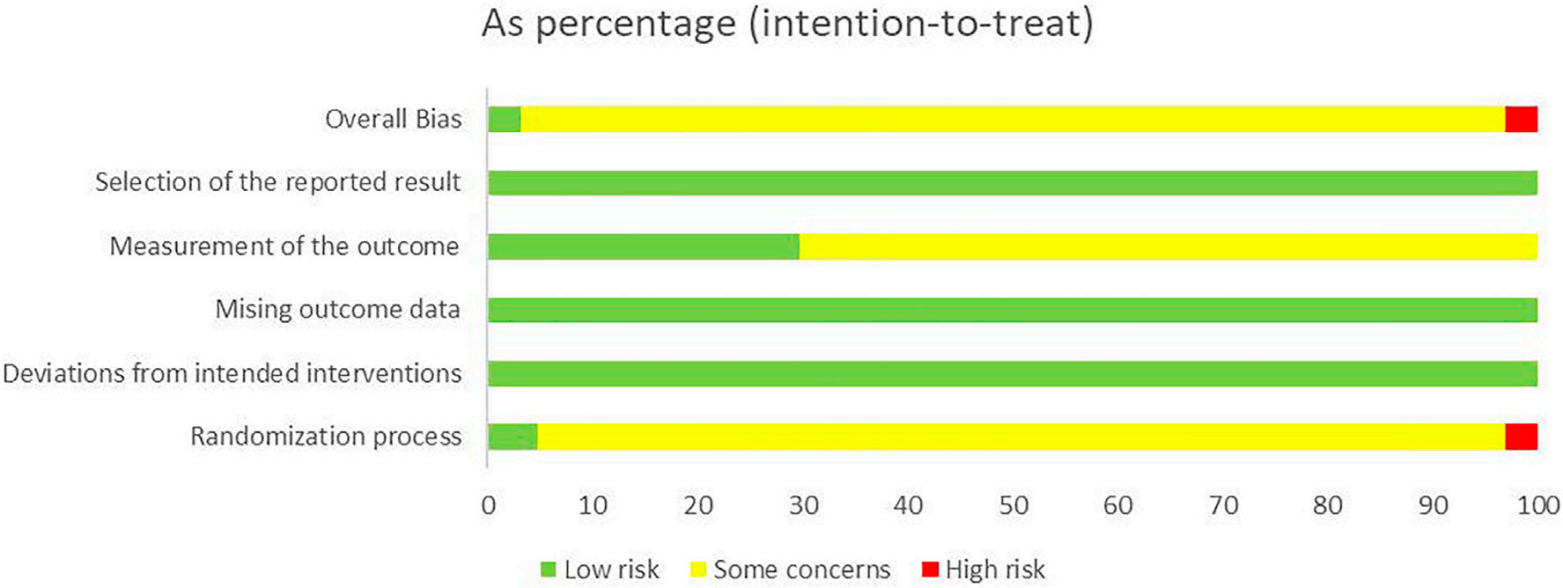
Risk-of-bias summary.

We used the RoB 2 to conduct a quality evaluation. Three studies were assessed as “low risk” (Zhu and Liu, 2014; Kong, 2015; Song et al., 2019) and two studies were “high risk” for the randomization process because of their incorrect method of random sequence generation (Tian et al., 2019; Deng and Chen, 2021). All trials were rated to have low risk of bias for deviations from intended interventions, missing outcome data, and selection of the reported result. Nineteen studies were evaluated as “low risk” in measurement of the outcome (Han et al., 2012; Li, 2013; Lin et al., 2013; Chen et al., 2014; Gao et al., 2014; Zhu and Liu, 2014; Song and Chai, 2015; Zhang and Wang, 2015; Zhuang, 2016; Diao et al., 2017; Lei et al., 2017; Lv et al., 2017; Chen, 2019; Sheng et al., 2019; Song et al., 2019; Zhao et al., 2019; Yang, 2020; Yang et al., 2020; Yuan and Zhao, 2020), and the remaining were assessed as “some concerns.” In general, most studies were classified as “some concerns.” Further details of the risk of bias assessment are shown in Figures 3 and 4. In addition, although blinding was not implemented and applicable for subjects in most studies, the lack of blinding was unlikely to have influenced the assessment of primary outcome indicators.

## Outcomes

### The Clinical Effective Rate

The clinical effective rate was deemed as the primary outcome. A total of 46 RCTs (Qi et al., 2011; Wu and Ru, 2011; Sun et al., 2012; Wu, 2012; Gao et al., 2014; Yang et al., 2014; Zhang et al., 2014; Huang, 2015; Kong, 2015; Wu, 2015; Ma, 2016; Wu and Ren, 2016; Xi et al., 2016; Xie, 2016; Li, 2017; Niu et al., 2017; Pan et al., 2017; Wang et al., 2017; Xia and Xie, 2017; Xu, 2017; Zhou et al., 2017; Fan et al., 2018; Meng and Xie, 2018; Wang, 2018; Zhou, 2018; Chen et al., 2019; Liu and Qin, 2019; Shang and Zhang, 2019; Tian et al., 2019; Wang, 2019; Wang, 2019; Wang et al., 2019; Yang and Zhang, 2019; Yin, 2019; Cai, 2020; Ding and Lu, 2020; Sun et al., 2020; Wang et al., 2020; Wei and Huang, 2020; Xiao et al., 2020; Xin, 2020; Zhang et al., 2020; Zheng et al., 2020; Deng and Chen, 2021; Qiu, 2021; Zhang, 2021) of six CHIs reported the clinical effective rate. In the pairwise meta-analysis, there was no significant heterogeneity in the pooled analysis of all included studies (*p* = 1.000, *I* ^2^ = 0.0%). The results of the heterogeneity are shown in Table 2. According to the network of comparisons in Table 2, there were 21 comparisons, and XBJ, TRQ, RDN, and SM combined with WM improved the clinical effective rate more significantly than WM alone (XBJ + WM: OR = 0.24, 95% CI: 0.19, 0.30; TRQ + WM: OR = 0.22, 95% CI: 0.12, 0.37; RDN + WM: OR = 0.29, 95% CI: 0.04, 0.94; and SM + WM: OR = 0.27, 95% CI: 0.08, 0.63). However, the results showed no significant differences between XYP and SF combined with WM than WM alone (XYP + WM: OR = 0.30, 95% CI: 0.03, 1.09 and SF + WM: OR = 0.47, 95% CI: 0.09, 1.36).

**TABLE 2:** ORs with 95% CIs of the clinical effective rate.

WM	*p* = 1.000, *I* ^2^ = 0.0%	*p* = 1.000, *I* ^2^ = 0.0%				*p* = 0.863, *I* ^2^ = 0.0%
0.24 (0.19, 0.30)	**XBJ + WM**					
0.22 (0.12, 0.37)	0.94 (0.49, 1.62)	**TRQ + WM**				
0.29 (0.04, 0.94)	1.23 (0.17, 4.03)	1.42 (0.18, 4.94)	**RDN + WM**			
0.30 (0.03, 1.09)	1.26 (0.11, 4.62)	1.45 (0.12, 5.54)	1.93 (0.07, 9.95)	**XYP + WM**		
0.47 (0.09, 1.36)	1.96 (0.37, 5.86)	2.27 (0.38, 7.33)	2.99 (0.22, 13.72)	3.99 (0.19, 20.87)	**SF + WM**	
0.27 (0.08, 0.63)	1.13 (0.33, 2.71)	1.31 (0.34, 3.41)	1.72 (0.17, 7.21)	2.28 (0.16, 11.25)	0.94 (0.12, 3.60)	**SM + WM**

**FIGURE 5:**
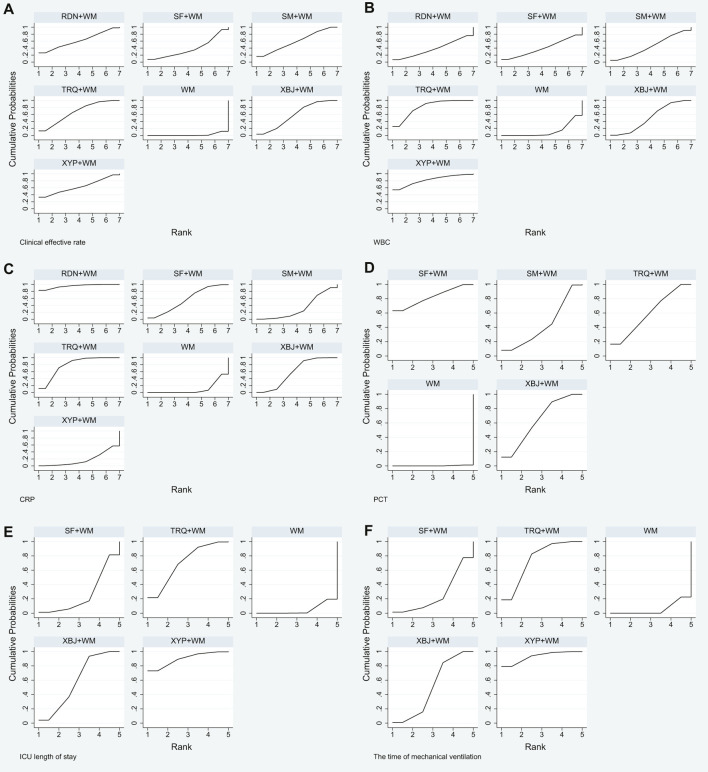
Plot of surface under the cumulative ranking curve (SUCRA) for all different outcomes. **(A)** Clinical effective rate; **(B)** the level of WBC; **(C)** the level of CRP; **(D)** the level of PCT; **(E)** ICU length of stay; and **(F)** the time of mechanical ventilation.

In Table 8 and Figure 5, the SUCRA values suggested that TRQ + WM was the optimal treatment, XYP + WM was the second, and RDN + WM was the third.

### The Level of WBC

As the other dominating outcome, WBC was estimated in 26 RCTs (Qi et al., 2011; Sun et al., 2012; Li, 2013; Gao et al., 2014; Yang et al., 2014; Zhang et al., 2014; Huang, 2015; Kong, 2015; Wu and Ren, 2016; Xi et al., 2016; Xie, 2016; Zhuang, 2016; Diao et al., 2017; Lei et al., 2017; Lv et al., 2017; Pan et al., 2017; Xu, 2017; Fan et al., 2018; Chen, 2019; Chen et al., 2019; Liu and Qin, 2019; Shang and Zhang, 2019; Yang and Zhang, 2019; Cai, 2020; Sun et al., 2020; Zhang, 2021). The heterogeneity results of the pairwise meta-analysis are shown in Table 3. According to Table 3, the CHIs like XBJ and TRQ combined with WM (XBJ + WM: SMD = −0.87, 95% CI: 1.37, −0.36 and TRQ + WM: SMD = −1.53, 95% CI: 2.20, −0.87) had better clinical effective rate than WM alone, and the differences among the above interventions were statistically significant. However, the rest of the CHIs including RDN, XYP, SF, and SM combined with WM did not perform more outstanding than WM alone. In addition, in terms of Table 3, there were no significant differences between each comparison of different types of CHIs.

**TABLE 3:** SMDs with 95% CIs of the level of WBC.

WM	*p* = 0.000, *I* ^2^ = 96.3%	*p* = 0.000, *I* ^2^ = 94.3%				*p* = 0.312, *I* ^2^ = 2.1%
−0.87 (−1.37, −0.36)	**XBJ + WM**					
−1.53 (−2.20, −0.87)	−0.68 (−1.50, 0.16)	**TRQ + WM**				
−0.51 (−2.40, 1.38)	0.36 (−1.60, 2.31)	1.023 (−0.97, 3.02)	**RDN + WM**			
−1.84 (−3.72, 0.04)	−0.97 (−2.92, 0.97)	−0.31 (−2.30, 1.69)	−1.33 (−3.99, 1.33)	**XYP + WM**		
−0.56 (−2.43, 1.30)	0.31 (−1.63, 2.23)	0.98 (−1.01, 2.95)	−0.05 (−2.71, 2.61)	1.28 (−1.37, 3.93)	**SF + WM**	
−0.74 (−2.07, 0.59)	0.13 (−1.30, 1.55)	0.80 (−0.69, 2.28)	−0.23 (−2.54, 2.08)	1.10 (−1.20, 3.40)	−0.18 (−2.47, 2.11)	**SM + WM**

Based on the ranking analysis, XYP + WM attained the first rank. TRQ + WM was the second, and XBJ + WM was the third (Table 8 and Figure 5).

### The Level of CRP

CRP was tested in 30 RCTs (Qi et al., 2011; Sun et al., 2012; Gao et al., 2014; Zhang et al., 2014; Kong, 2015; Zhang and Wang, 2015; Xi et al., 2016; Xie, 2016; Zhuang, 2016; Diao et al., 2017; Lei et al., 2017; Lv et al., 2017; Pan et al., 2017; Xia and Xie, 2017; Xu, 2017; Fan et al., 2018; Chen, 2019; Chen et al., 2019; Liu and Qin, 2019; Shang and Zhang, 2019; Song et al., 2019; Tian et al., 2019; Yang and Zhang, 2019; Cai, 2020; Ding and Lu, 2020; Sun et al., 2020; Xiao et al., 2020; Yang, 2020; Yuan and Zhao, 2020; Zhang, 2021) involved seven interventions. The heterogeneity results of the pairwise meta-analysis are shown in Table 4. Four of them were noticeably better than WM treatment alone for decreasing the level of CRP, as XBJ + WM (SMD = −1.62, 95% CI: 2.06, −1.18), TRQ + WM (SMD = −2.15, 95% CI: 2.93, −1.36), RDN + WM (SMD = −3.72, 95% CI: 5.19, −1.35), and SF + WM (SMD = −1.52, 95% CI: 2.87, −0.17) were remarkable among them compared with WM alone. What is more, based on WM, TRQ and RDN had a more excellent performance in decreasing CRP than XBJ (XBJ + WM vs TRQ + WM: SMD = −0.53, 95% CI: 1.43, −0.53 and XBJ + WM vs RDN + WM: SMD = −1.66, 95% CI: 3.63, −1.66), and the results between the rest of the comparisons of different CHIs showed no significant differences.

**TABLE 4:** SMDs with 95% CIs of the level of CRP.

WM	*p* = 0.000, *I* ^2^ = 97.1%	*p* = 0.000, *I* ^2^ = 85.7%			*p* = 0.000, *I* ^2^ = 98.2%	*p* = 0.000, *I* ^2^ = 92.5%
**−1.62 (−2.06, −1.18)**	**XBJ + WM**					
**−2.15 (−2.93, −1.36)**	−**0.53** (−**1.43,** −**0.53** )	**TRQ + WM**				
**−3.27 (−5.19, −1.35)**	−**1.66** (−**3.63,** −**1.66**)	−1.13 (−3.31, 0.95)	**RDN + WM**			
−0.13 (−2.06, 1.80)	1.49 (−0.49, 3.47)	2.02 (−0.07, 4.10)	3.14 (0.42, 5.86)	**XYP + WM**		
**−1.52 (−2.87, −0.17)**	0.10 (−1.32, 1.52)	0.63 (−0.94, 2.19)	1.75 (−0.60, 4.10)	−1.39 (−3.75, 0.96)	**SF + WM**	
−0.76 (−2.12, 0.60)	0.86 (−0.57, 2.28)	1.39 (−0.19, 2.96)	2.51 (0.16, 4.87)	−0.63 (−2.99, 1.73)	0.76 (−1.16, 2.68)	**SM + WM**

Significant effects are printed in bold.

The SUCRA mentioned above was also affirmed, and RDN + WM was the best choice, followed by TRQ + WM and XBJ + WM (Table 8 and Figure 5).

### The Level of PCT

The potency of decreasing the level of PCT was assessed. The heterogeneity results of the pairwise meta-analysis are shown in Table 5. Four interventions with 22 RCTs (Chen et al., 2014; Gao et al., 2014; Kong, 2015; Wu and Ren, 2016; Pan et al., 2017; Xia and Xie, 2017; Fan et al., 2018; Chen, 2019; Liu and Qin, 2019; Sheng et al., 2019; Song et al., 2019; Tian et al., 2019; Wang, 2019; Yang and Zhang, 2019; Zhao et al., 2019; Cai, 2020; Xiao et al., 2020; Xin, 2020; Yang, 2020; Yang et al., 2020; Yuan and Zhao, 2020; Zhang, 2021) had data in contrast with WM, shown in Table 5. The results revealed that all CHIs involved combined with WM were advantageous in decreasing PCT compared to WM alone (XBJ + WM: SMD = −2.19, 95% CI: 2.67, −1.70; TRQ + WM: SMD = −2.12, 95% CI: 3.27, −0.96; SF + WM: SMD = −2.84, 95% CI: 4.83, −0.86; and SM + WM: SMD = −1.69, 95% CI: 3.11, −0.27), and the differences among the abovementioned combinations were statistically significant.

**TABLE 5:** SMDs with 95% CIs of the level of PCT.

WM	*p* = 0.000, *I* ^2^ = 98.2%	*p* = 0.085, *I* ^2^ = 59.4%		*p* = 0.000, *I* ^2^ = 97.5%
**−2.19 (−2.67, −1.70)**	**XBJ + WM**			
**−2.12 (−3.27, −0.96)**	0.07 (−1.19, 1.32)	**TRQ + WM**		
**−2.84 (−4.83, −0.86)**	−0.66 (−2.70, 1.39)	−0.73 (−3.03, 1.57)	**SF + WM**	
**−1.69 (−3.11, −0.27)**	0.50 (−1.00, 1.99)	0.43 (−1.40, 2.25)	1.16 (−1.29, 3.60)	**SM + WM**

Significant effects are printed in bold.

Treatment ranking based on SUCRA values, from largest to smallest, were as follows: SF + WM, XBJ + WM, TRQ + WM, SM + WM, and WM. The details are depicted in Table 8 and Figure 5.

### ICU Length of Stay

Twenty-two RCTs (Han et al., 2012; Li, 2013; Lin et al., 2013; Chen et al., 2014; Gao et al., 2014; Yang et al., 2014; Zhang et al., 2014; Zhu and Liu, 2014; Song and Chai, 2015; Ma, 2016; Lei et al., 2017; Lv et al., 2017; Pan et al., 2017; Wang, 2018; Zhou, 2018; Chen et al., 2019; Sheng et al., 2019; Wang et al., 2019; Zhao et al., 2019; Wei and Huang, 2020; Xiao et al., 2020; Deng and Chen, 2021) with five treatments reported the ICU length of stay. The heterogeneity results of the pairwise meta-analysis are shown in Table 6. As shown in Table 6, XBJ + WM (SMD = −1.02, 95% CI: 1.33, −0.72), TRQ + WM (SMD = −1.24, 95% CI: 2.16, −0.31), and XYP + WM (SMD = −1.78, 95% CI: 3.07, −0.49) were more effective than WM alone. However, the results showed no significant difference in most cases.

**TABLE 6:** SMDs with 95% CIs of ICU length of stay.

WM	*p* = 0.000, *I* ^2^ = 78.7%	*p* = 0.003, *I* ^2^ = 88.3%		*p* = 0.430, *I* ^2^ = 0.0%
**−1.02 (−1.33, −0,72)**	**XBJ + WM**			
**−1.24 (−2.16, −0.31)**	−0.22 (−1.19, 0.76)	**TRQ + WM**		
**−1.78 (−3.07, −0.49)**	−0.76 (−2.09, 0.57)	−0.54 (−2.14, 1.05)	**XYP + WM**	
−0.40 (−1.33, 0.53)	0.62 (−0.36, 1.60)	0.84 (−0.48, 2.15)	1.38 (−0.21, 2.97)	**SF + WM**

Significant effects are printed in bold.

Based on the ranking analysis, XYP + WM attained the first rank. TRQ + WM was the next, and XBJ + WM was the third (Table 8 and Figure 5).

### The Time of Mechanical Ventilation

In terms of the time of mechanical ventilation, five treatments with 17 RCTs (Wu and Ru, 2011; Han et al., 2012; Li, 2013; Chen et al., 2014; Gao et al., 2014; Yang et al., 2014; Zhu and Liu, 2014; Song and Chai, 2015; Ma, 2016; Lv et al., 2017; Pan et al., 2017; Wang, 2018; Liu and Qin, 2019; Wang et al., 2019; Wei and Huang, 2020; Yang, 2020; Deng and Chen, 2021) were compared with WM. The heterogeneity results of the pairwise meta-analysis are shown in Table 7. As seen in Table 7, three CHIs combined with WM (XBJ + WM: SMD = −1.72, 95% CI: 2.26, −1.17; TRQ + WM: SMD = −2.42, 95% CI: 3.56, −1.28; and XTP + WM: SMD = −3.41, 95% CI: 5.40, −1.43) had excellent performance in decreasing the time of mechanical ventilation compared to WM alone, and the results were statistically significant.

**TABLE 7:** SMDs with 95% CIs of the time of mechanical ventilation.

WM	*p* = 0.000, *I* ^2^ = 90.2%	*p* = 0.000, *I* ^2^ = 94.3%		
**−1.72 (−2.26, −1.17)**	**XBJ + WM**			
**−2.42 (−3.56, −1.28)**	−0.71 (−1.97, 0.56)	**TRQ + WM**		
**−3.41 (−5.40, −1.43)**	−1.70 (−3.76, 0.36)	−0.99 (−3.29, 1.30)	**XYP + WM**	
−0.77 (−2.73, 1.21)	0.95 (−1.09, 3.00)	1.66 (−0.61, 3.94)	2.65 (−0.14, 5.45)	**SF + WM**

Significant effects are printed in bold.

Results of ranking analysis manifested that XYP + WM was efficacious in decreasing the time of mechanical ventilation. Other beneficial treatments were TRQ + WM and XBJ + WM (Table 8 and Figure 5).

**TABLE 8:** SUCRA results of the outcomes.

	Clinical effective rate (%)	The level of WBC (%)	The level of CRP (%)	The level of PCT (%)	ICU length of stay (%)	The time of mechanical ventilation (%)
XBJ + WM	58.5	50.9	58.9	63.8	58.6	50.3
TRQ + WM	66.4	80.9	78.9	60.0	70.4	74.6
RDN + WM	61.6	38.2	95.1	–	–	–
XYP + WM	63.4	82.0	18.1	–	89.7	92.9
SF + WM	38.7	39.8	56.3	82.2	26.4	26.6
SM + WM	59.4	45.8	32.7	43.7	–	–
WM	2.1	12.3	10.0	0.3	4.7	5.6

### Cluster Analysis

**FIGURE 6:**
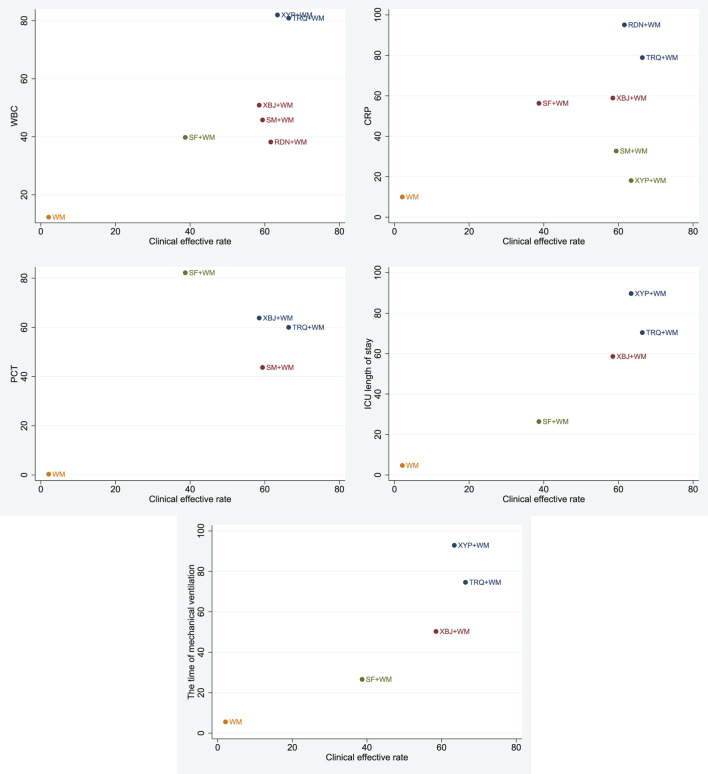
Cluster analysis plot for six outcomes. Note: interventions with the same color belong to the same cluster, and interventions located in the upper right corner indicate optimal therapy for two different outcomes.

A cluster analysis was performed on the primary outcome and secondary outcomes for the seven treatments. As shown in Figure 6, in terms of the clinical effective rate and the level of WBC, the clinical effective rate and ICU length of stay, and the clinical effective rate and the time of mechanical ventilation, TRQ and XYP combined with WM were similarly superior. According to the clinical effective rate and the level of CRP, TRQ and RDN combined with WM were more beneficial. About the clinical effective rate and the level of PCT, TRQ and XBJ combined with WM were preferred.

### ADRs/ADEs

Among 64 RCTs, a total of 18 RCTs reported the ADRs/ADEs of interventions. There were 14 RCTs (Gao et al., 2014; Zhang et al., 2014; Ma, 2016; Wu and Ren, 2016; Pan et al., 2017; Meng and Xie, 2018; Zhou, 2018; Shang and Zhang, 2019; Song et al., 2019; Wang, 2019; Wang et al., 2019; Xin, 2020; Deng and Chen, 2021; Qiu, 2021) involving 1,095 participants with XBJ group that reported ADRs, including headache and dizziness (32 cases in 10 RCTs), diarrhea (10 cases in 7 RCTs), nausea and vomiting (4 cases in 3 RCTs), chest discomfort or dyspnea (4 cases in 4 RCTs), itchy skin or rash (9 cases in 6 RCTs), and myelosuppression (4 cases in 4 RCTs). Three RCTs (Huang, 2015; Xi et al., 2016; Zhang, 2021) reported ADRs/ADEs of TRQ, one of the RCTs reported two cases of nausea and vomiting and one case of rash. Another two RCTs did not report ADRs/ADEs. In addition, only one RCT (Sun et al., 2012) mentioned the ADRs of RDN and one case reported thirst. The rest of the included studies did not provide information on any ADRs/ADEs. All of the symptoms were alleviated after corresponding treatment and did not influence the RCTs.

### Sensitivity Analysis

To assess the robustness and reliability of the primary outcome results, a sensitivity analysis was conducted based on the results of quality assessments of studies. Two studies were excluded for their high risk of bias (Tian et al., 2019; Deng and Chen, 2021), and the remaining 44 studies conducted a network meta-analysis again. Results did not show relevant deviations compared with the original network meta-analysis. The Bayesian ranking results from high to low for clinical effective rate were TRQ (66.3%), XYP (62.9%), RDN (61.4%), XBJ (59.1%), SF (58.7%), SM (39.5%), and WM (2.0%), respectively. The sensitivity analysis showed that the results of clinical effective rate were robust and reliable.

### Funnel Plot Characteristics

**FIGURE 7:**
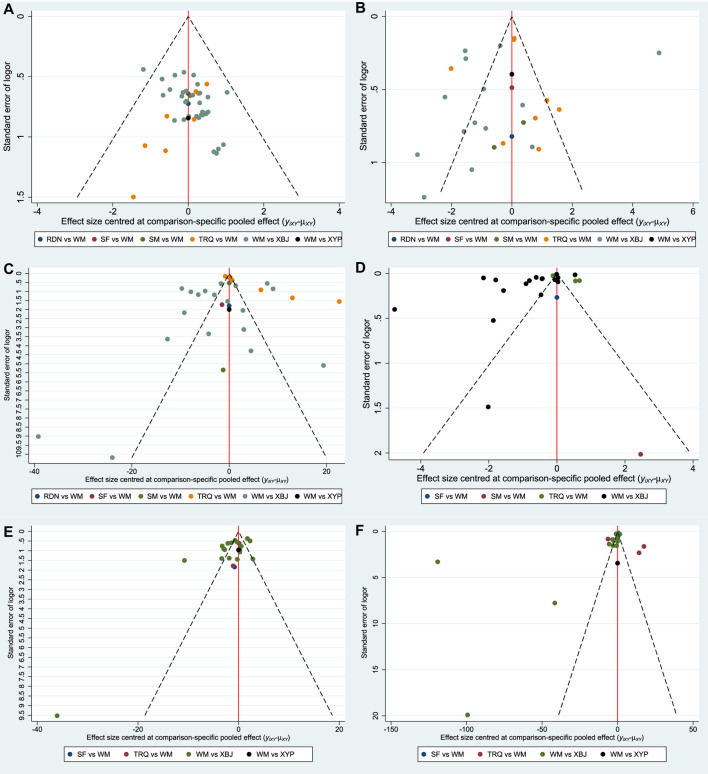
Funnel plot. **(A)** Clinical effective rate; **(B)** the level of WBC; **(C)** the level of CRP; **(D)** the level of PCT; **(E)** ICU length of stay; and **(F)** the time of mechanical ventilation.

Comparison-adjusted funnel plots for different outcomes are displayed in Figure 7. There were three funnel plots of outcomes that were generally symmetrical visually, including the clinical effective rate, ICU length of stay, and the time of mechanical ventilation. Therefore, they had no publication bias. Among the remaining outcomes, the funnel plots were not symmetrically visual, which revealed that there were small sample size and publication bias.

## Discussion

A total of 64 RCTs involving 5,904 participants were included. Six CHIs were identified in the treatment of SP, including XBJ, TRQ, RDN, XYP, SF, and SM. The Mantel-Haenszel random-effects model was used for the meta-analysis because of the heterogeneity. The assumption of consistency between direct and indirect evidence was not utilized due to non-closed loops in this NMA. Six interested outcomes were identified in this network meta-analysis, including clinical effective rate; the time of mechanical ventilation; ICU length of stay; and serum levels of WBC, CRP, and PCT. The results indicated that XBJ, TRQ, RDN, and SM combined with WM had a superior effect than WM alone in terms of the outcome of clinical effective rate. Based on SUCRA values, TRQ combined with WM ranked the highest in improving the clinical effective rate, second in four different outcomes, and third in only one. Similarly, XYP combined with WM ranked second in the clinical effective rate but ranked the highest in three outcomes, which were the level of WBC, ICU length of stay, and the time of mechanical ventilation. What is more, according to the cluster analysis, TRQ and XYP combined with WM were similarly superior in terms of the clinical effective rate and the level of WBC, the clinical effective rate and ICU length of stay, and the clinical effective rate and the time of mechanical ventilation. Therefore, TRQ and XYP combined with WM were worth paying more attention for SP in adults. However, only one RCT of XYP involving 68 participants was included in our study. Taking into account the small sample size, the strength of evidence for this result may be reduced.

As for safety, less than 30% (18 RCTs) of the included studies reported ADRs/ADEs, including 14 RCTs of XBJ, 3 RCTs of TRQ, and 1 RCT of RDN. The ADRs/ADEs mainly included headache and dizziness, nausea and vomiting, diarrhea, chest discomfort or dyspnea, and itchy skin or rash. It is noteworthy that headache and dizziness occurred most frequently among the abovementioned ADRs/ADEs. Though all the ADRs/ADEs were mild and can be relieved by themselves, no RCTs reported the rate of ADRs/ADEs comparing CHIs combined with WM and WM alone. Hence, we could not draw a certain conclusion that combining CHIs with WM will not increase the ADRs/ADEs of the patients. Hopefully, further studies especially clinical trials should pay more attention to these ADRs/ADEs of CHIs, and more studies are needed to determine the safety of CHIs combined with WM for SP.

SP is a complex and refractory disease. The causative pathogen produces an excessive inflammatory response with high levels of anti-inflammatory cytokines. High levels of anti-inflammatory cytokines are initially detected in the plasma and the lungs. These high levels of anti-inflammatory cytokines are associated with ICU admission and mortality (Ramírez et al., 2011). As the present major treatment, adequate initial antibiotics can cause serious drug resistance and did not reduce the mortality rate of patients admitted to the ICU for SP (Garnacho-Montero et al., 2018). Co-adjuvant therapies such as CHIs become more attractive. CHIs combined with WM for SP exhibited a better performance in improving the clinical effective rate and the inflammatory indicators (WBC, CRP, and PCT) and decreasing the ICU length of stay and the time of mechanical ventilation, which may provide the solution for the above problems. Additionally, “Huang di Nei Jing” recorded that “keep healthy, do not be evil.” SP belongs to deficiency in origin and excess in superficiality. Antipyretic CHIs such as TRQ and XYP can relieve fever, become anti-inflammatory, and increase antibiotic sensitivity (Pan, 2015). Restorative CHIs such as SF can lower the pro-inflammatory cytokines and shorten the time of mechanical ventilation, ICU length of stay, the application time of vasoactive drugs, and even the mortality (Zhang and Wang, 2015; Lv et al., 2017). All the above advantages were closely related to the ingredients within them.

TRQ is a traditional Chinese medicine (TCM) consisting of five herbals extracts: Scutellariae Radix, bear bile powder, cornugorais, Lonicerae japonicae flos, and Forsythiae fructus. Based on the theory of traditional Chinese medicine, Scutellariae Radix has a bitter taste and the great effects of clearing heat, drying moisture, and detoxification. Bear bile power has the functions of reducing heat, spasmolysis, and detoxification. Cornugorais could enter the liver meridian and has significant antipyretic and sedative effects. Lonicerae japonicae flos can clear heat and detoxify and dispel the wind. When it is compatible with Forsythiae fructus, its effect will be more significant. In general, TRQ formula clears heat, detoxifies, and removes phlegm according to the traditional Chinese medicine theory. In terms of modern pharmacology, recent *in vivo* experiments had demonstrated that TRQ had antibacterial and antiviral effects. In some *in vitro* antibacterial tests, TRQ also showed a strong effect against some bacteria, such as *Streptococcus pneumoniae*, *Staphylococcus aureus*, methicillin-resistant *Staphylococcus aureus* (MRSA), and *Haemophilus influenzae*, and has markedly strengthened the antibacterial effect of antibiotics (Yang et al., 2018; Wang et al., 2020). Nevertheless, utilizing TRQ had some concerns. As one of the compositions of Tanreqing, bear bile powder is derived from *Selenarctos thibetanus Cuvier.* In China, the law has prohibited to get bear bile powder from hunting *S. thibetanus Cuvier*. The bear bile powders used as medicine are mainly derived from artificial feeding bears. What is more, only the farms which have gotten the license of domesticating and breeding wildlife under special state protection are qualified to feed *S. thibetanus Cuvier* (Author Anonymous, 2004; Author Anonymous, 2013; Author Anonymous, 2015). In addition, the technology of getting bear bile powder is mature and safe by surgical drainage from the bears’ gall bladder. But even so, in consideration of protecting endangered animals and dealing with increased demand of medicine, studying the alternatives is urged. XYP is mainly composed of andrographolide sulfonate. The main active components of SF include ginsenoside and aconite total alkaloids.

There are three advantages that could enhance the credibility of this study. First, to the best of our knowledge, this is the first NMA to compare the effects of different CHIs and rank them for the treatment of SP. Secondly, these results may be helpful to clinicians to make a better choice for the treatment of SP. More importantly, in addition to the clinical phenomena and efficacy, the ICU length of stay, the time of mechanical ventilation, and inflammatory indicators were also analyzed. Inflammatory indicators are more essential to SP because of their relativity to the SP’s pathophysiology. What is more, inflammatory indicators are also relative to the mechanism of drug effect. Critically patients are complex and under many different interventions such as mechanical ventilation. ICU length of stay and the time of mechanical ventilation can not only reflect clinical effects from the side but also reflect economic benefits.

### Limitation

This study also has some limitations. First, all RCTs were carried out in China, and the data of clinical studies in other languages were lacking. Second, the qualities of included RCTs in this study were not high. About 58% of RCTs described the method of generating random sequences. Only two RCTs described information of allocation concealment and one RCT accurately set blinding. Third, there was a lack of large sample directly comparing the two injections. The difference among the sample sizes of different injections would also reduce the strength of the evidence for the results. It is necessary to conduct a subgroup analysis based on background diseases, different types of pneumonia, duration of treatment, and Western medicine treatment measure. However, except XBJ injection, the number of included studies for other CHIs was not enough to conduct a subgroup analysis. In addition, it is difficult to conduct a subgroup analysis as the currently included studies could not be accurately categorized based on the above variables. We hope that more RCTs could include only one background disease, one type of pneumonia (CAP, HAP, or VAP), one kind of antibiotics, and a fixed treatment duration in developing the inclusion criteria.

## Conclusion

In conclusion, the study found that the CHIs as a co-adjuvant therapy could be beneficial for patients with SP. TRQ + WM had preferable effects in improving the clinical effective rate of SP. XYP + WM was more effective in the perspective of reducing the level of WBC, the ICU length of stay, and the time of mechanical ventilation. So, considering both the clinical effective rate and other outcomes, TRQ + WM showed an outstanding improvement in patients with SP. However, because of the limitations of this study, the results should be verified by more high-quality and large-sample RCTs. Meanwhile, the safety of CHIs should be more monitored and reported.

## Data Availability

The original contributions presented in the study are included in the article/Supplementary Material; further inquiries can be directed to the corresponding author.
