# Supplementary material for: Comparative Efficacy of Chinese Herbal Injections for Treating Severe Pneumonia: A Systematic Review and Bayesian Network Meta-Analysis of Randomized Controlled Trials
Source: Front Pharmacol. 2022 Jan 10;12:743486. doi: 10.3389/fphar.2021.743486 (PMC8784988; doi:10.3389/fphar.2021.743486)
Supplement: Supplementary file 2 [file DataSheet1.ZIP › Figure 3-Risk of bias graph.pdf]

Study ID	D1	D2	D3	D4	D5	Overall
Qi F 2011	!	+	+	!	+	!
Li ZX 2017	!	+	+	!	+	!
Zhou SJ 2017	!	+	+	!	+	!
Yin ZM 2019	!	+	+	!	+	!
Shang HB 2019	!	+	+	!	+	!
Chen S 2019	!	+	+	!	+	!
Xin RR 2020	!	+	+	!	+	!
Xiao Q 2020	!	+	+	!	+	!
Chen JZ 2019	!	+	+	+	+	!
Meng SD 2018	!	+	+	!	+	!
Wang L 2019	!	+	+	!	+	!
Zhou ZY 2018	!	+	+	!	+	!
Wang LL 2020	!	+	+	!	+	!
Wang M 2017	!	+	+	!	+	!
Tian J 2019	-	+	+	!	+	-
Zhang SL 2014	!	+	+	!	+	!
Xie GL 2016	!	+	+	!	+	!
Qiu JN 2021	!	+	+	!	+	!
Xu M 2017	!	+	+	!	+	!
Ding ZP 2020	!	+	+	!	+	!
Sheng N 2019	!	+	+	+	+	!
Wang DL 2019	!	+	+	!	+	!
Wang L 2018	!	+	+	!	+	!
Wang ZW 2019	!	+	+	!	+	!
Wei SX 2020	!	+	+	+	+	!
Wu T 2016	!	+	+	!	+	!
Yang HW 2020	!	+	+	+	+	!
Yang TB 2020	!	+	+	+	+	!
Chen YJ 2014	!	+	+	+	+	!
Diao YF 2017	!	+	+	+	+	!
Zheng YN 2020	!	+	+	!	+	!
Zhu JJ 2014	+	+	+	+	+	+
Zhuang L 2016	!	+	+	+	+	!
Zhao YJ 2019	!	+	+	+	+	!
Pan XB 2017	!	+	+	!	+	!
Song Y 2019	+	+	+	+	+	+
Wu JH 2015	!	+	+	!	+	!
Song B 2015	!	+	+	+	+	!
Gao YQ 2014	!	+	+	+	+	!
Ma LH 2016	!	+	+	!	+	!
Niu LL 2017	!	+	+	!	+	!
Yuan HY 2020	!	+	+	+	+	!
Deng Z 2021	-	+	+	!	+	-
Han F 2012	!	+	+	+	+	!
Kong LY 2015	+	+	+	!	+	!
Zhang LL 2020	!	+	+	!	+	!
Cai CY 2020	!	+	+	!	+	!
Lei JP 2017	!	+	+	+	+	!
Huang SZ 2015	!	+	+	!	+	!
Wu XD 2012	!	+	+	!	+	!
Wu H 2011	!	+	+	!	+	!
Zhang HY 2021	!	+	+	!	+	!
Xi R 2016	!	+	+	!	+	!
Li J 2013	!	+	+	+	+	!
Sun JG 2020	!	+	+	!	+	!
Liu XL 2019	!	+	+	!	+	!
Sun GX 2012	!	+	+	!	+	!
Yang ZX 2014	!	+	+	!	+	!
Zhang LL 2015	!	+	+	+	+	!
Lv SJ 2017	!	+	+	+	+	!
Lin H 2013	!	+	+	+	+	!
Xia LF 2017	!	+	+	!	+	!
Fan XC 2018	!	+	+	!	+	!
Yang GL 2019	!	+	+	!	+	!

+ Low risk

! Some concerns

- High risk

D1 Randomisation process

D2 Deviations from the intended interventions

D3 Missing outcome data

D4 Measurement of the outcome

D5 Selection of the reported result
