# Supplementary figures and images for: Comparative Efficacy of Chinese Herbal Injections for Treating Severe Pneumonia: A Systematic Review and Bayesian Network Meta-Analysis of Randomized Controlled Trials
Source: Front Pharmacol. 2022 Jan 10;12:743486. doi: 10.3389/fphar.2021.743486 (PMC8784988; doi:10.3389/fphar.2021.743486)

Identification

Screening

Eligibility

Included

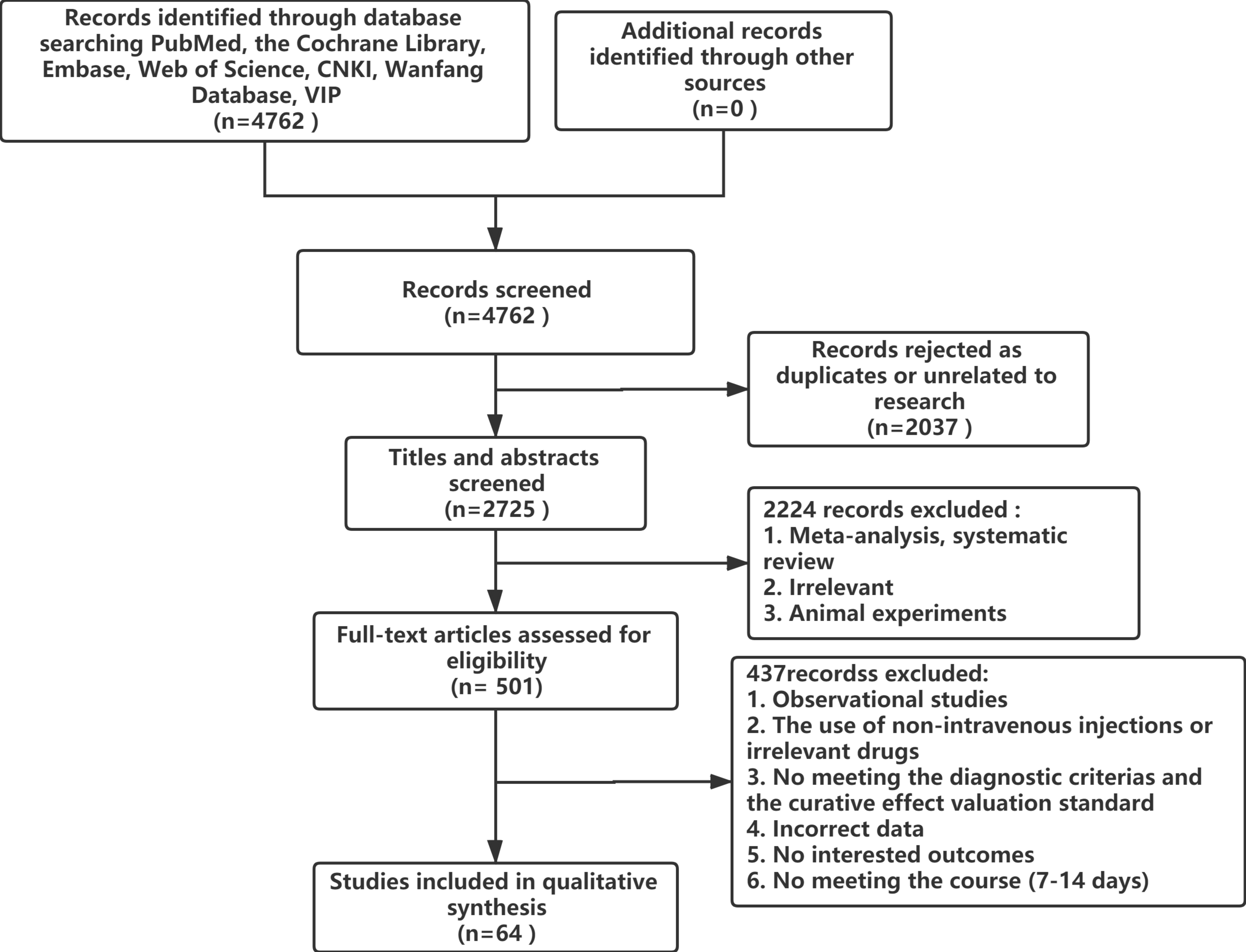

Supplement: Supplementary file 2 [file DataSheet1.ZIP › Figure 1-Flow diagram of study inclusion.pdf]

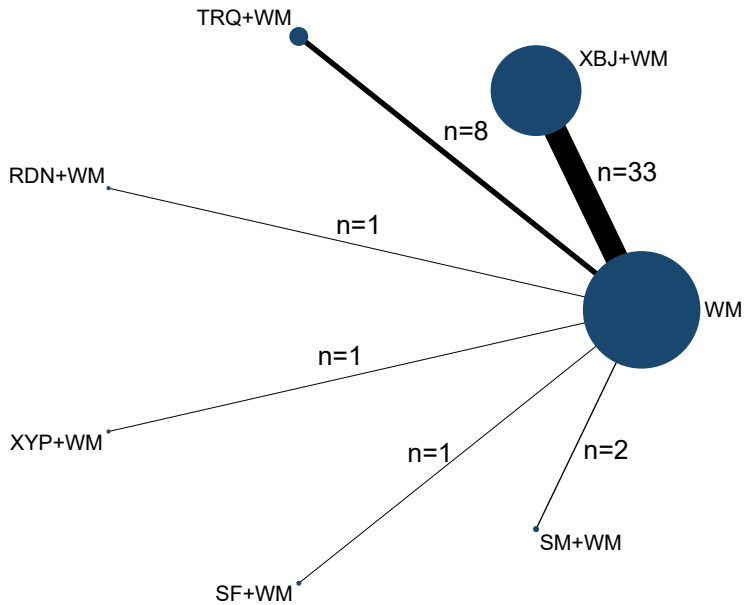

Supplement: Supplementary file 2 [file DataSheet1.ZIP › Figure 2-Network graph of the different outcomes/Figure 2-(A) Clinical effective rate.pdf]

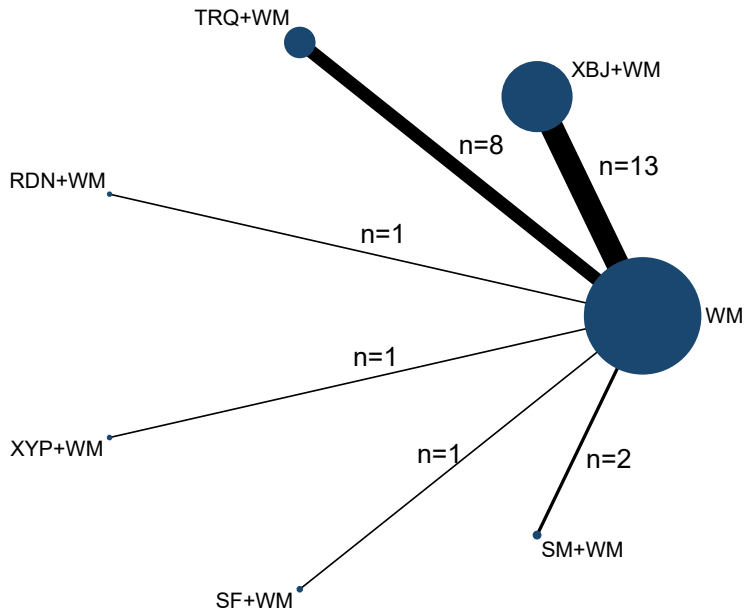

Supplement: Supplementary file 2 [file DataSheet1.ZIP › Figure 2-Network graph of the different outcomes/Figure 2-(B) WBC.pdf]

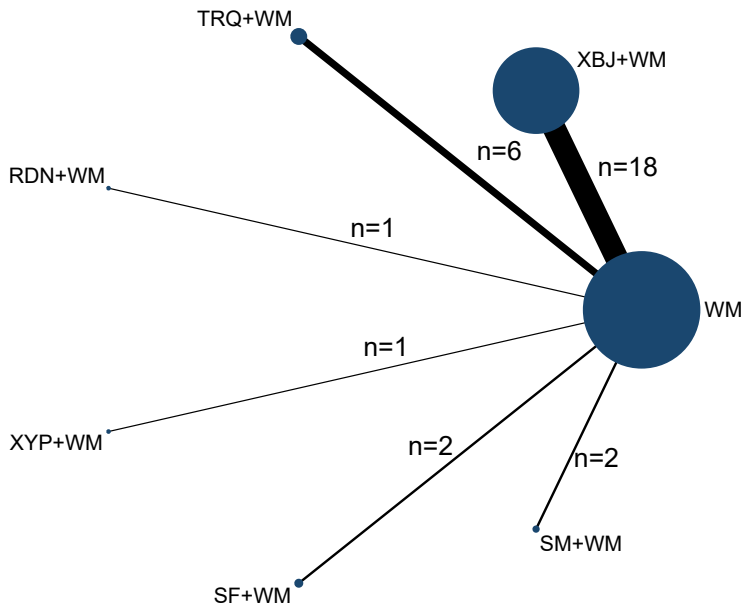

Supplement: Supplementary file 2 [file DataSheet1.ZIP › Figure 2-Network graph of the different outcomes/Figure 2-(C) CRP.pdf]

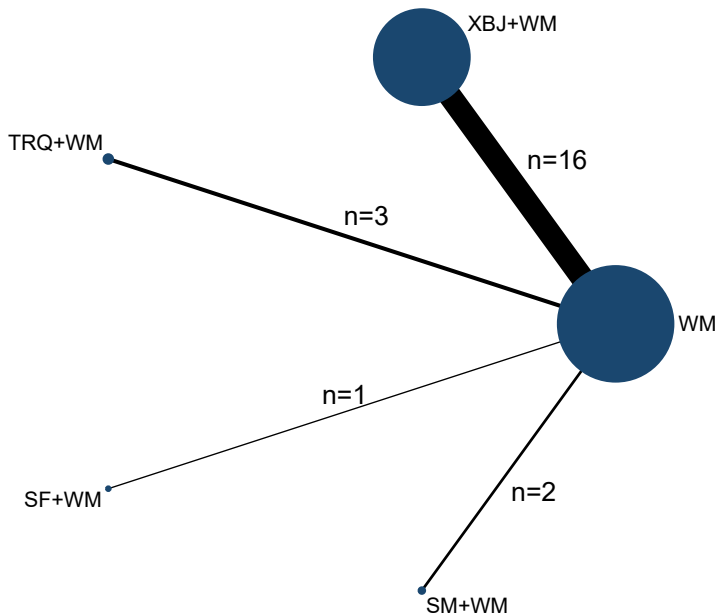

Supplement: Supplementary file 2 [file DataSheet1.ZIP › Figure 2-Network graph of the different outcomes/Figure 2-(D) PCT.pdf]

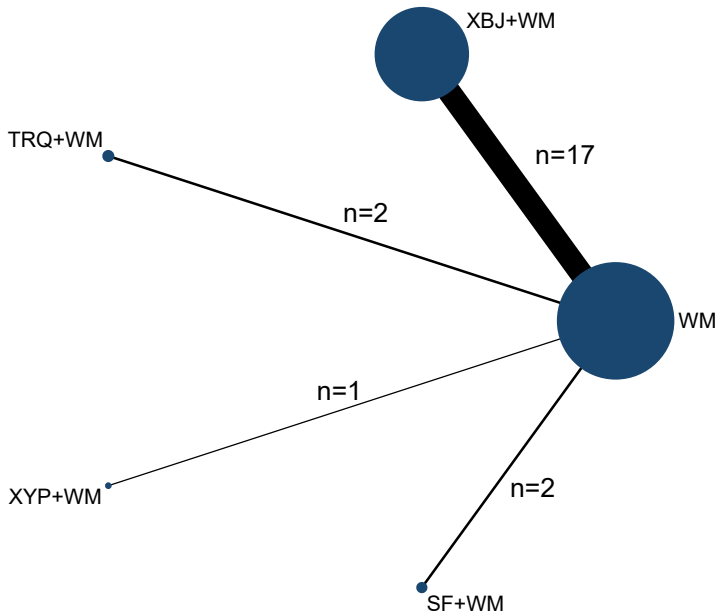

Supplement: Supplementary file 2 [file DataSheet1.ZIP › Figure 2-Network graph of the different outcomes/Figure 2-(E) ICU length of stay.pdf]

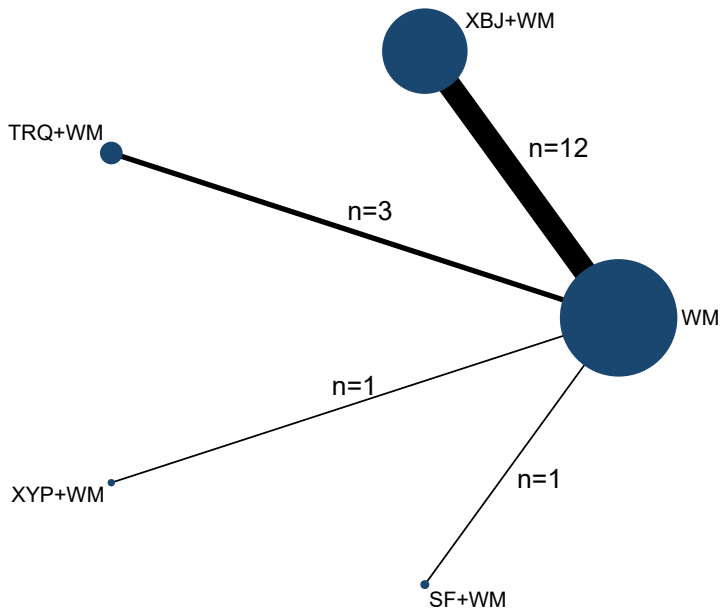

Supplement: Supplementary file 2 [file DataSheet1.ZIP › Figure 2-Network graph of the different outcomes/Figure 2-(F) The time of mechanical ventilation.pdf]

As percentage (intention-to-treat)

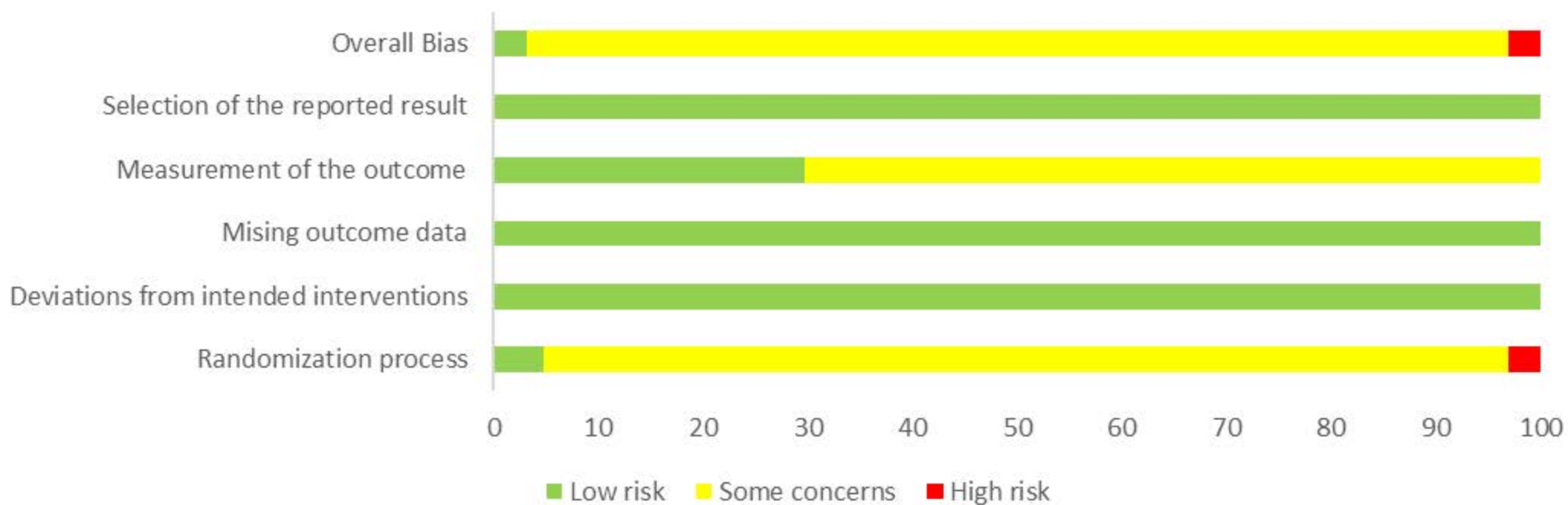

Supplement: Supplementary file 2 [file DataSheet1.ZIP › Figure 4-Risk of bias summary.pdf]

Cumulative Probabilities

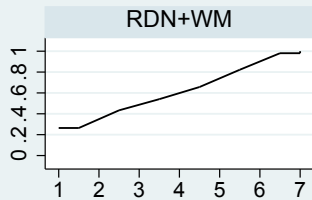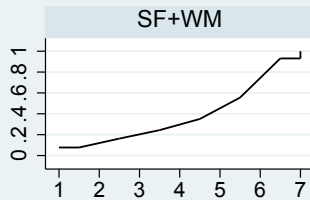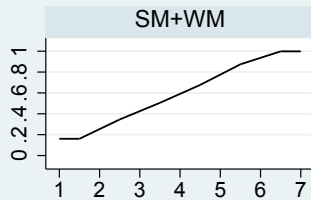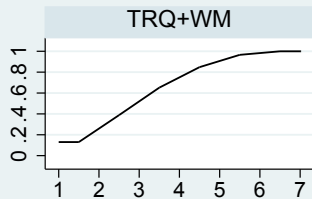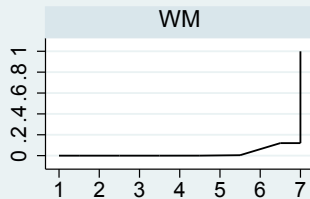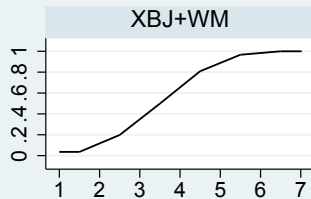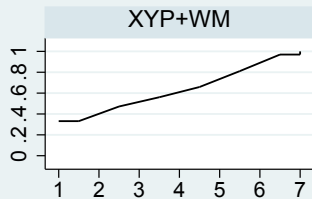

Rank

Clinical effective rate

Supplement: Supplementary file 2 [file DataSheet1.ZIP › Figure 5-Plot of SUCRA for all different outcomes/Figure 5-(A) Clinical effective rate.pdf]

Cumulative Probabilities

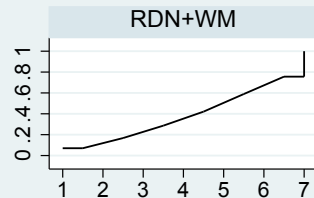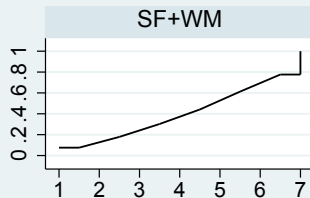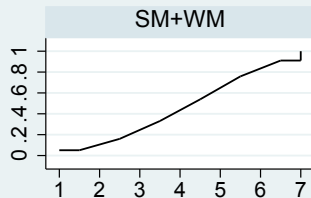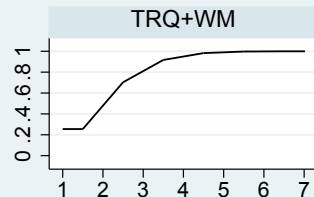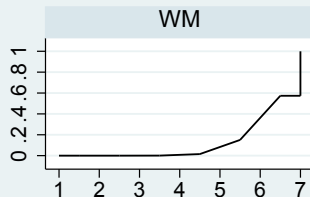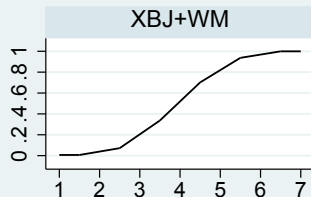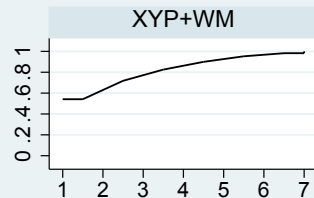

Rank

WBC

Supplement: Supplementary file 2 [file DataSheet1.ZIP › Figure 5-Plot of SUCRA for all different outcomes/Figure 5-(B) WBC.pdf]

Cumulative Probabilities

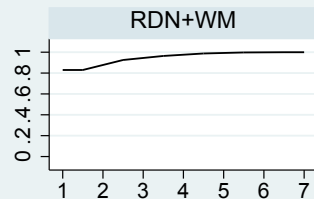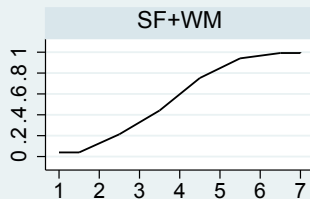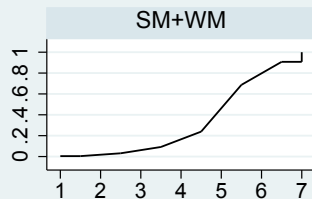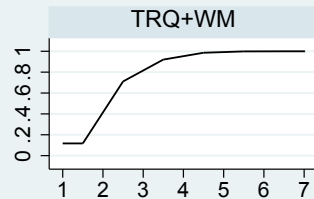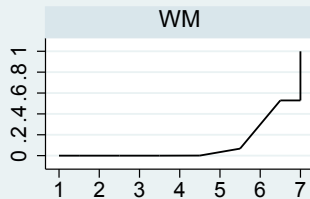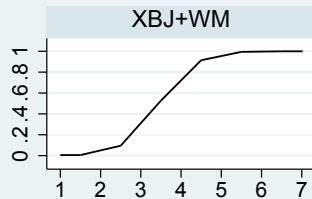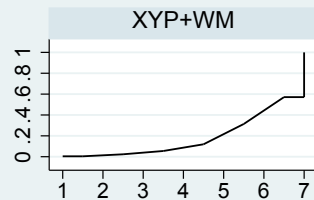

Rank

CRP

Supplement: Supplementary file 2 [file DataSheet1.ZIP › Figure 5-Plot of SUCRA for all different outcomes/Figure 5-(C) CRP.pdf]

Cumulative Probabilities

SF+WM

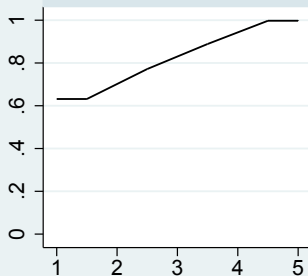

SM+WM

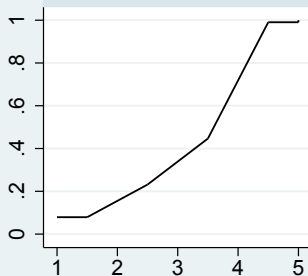

TRQ+WM

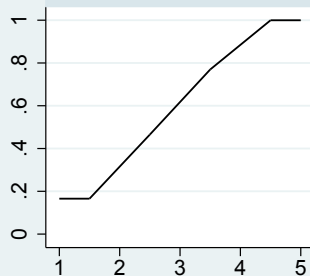

WM

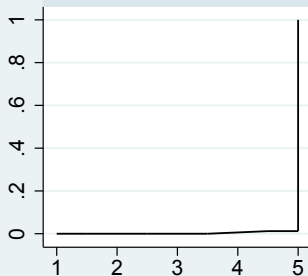

XBJ+WM

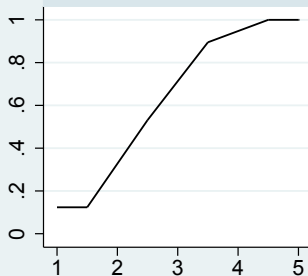

Rank

PCT

Supplement: Supplementary file 2 [file DataSheet1.ZIP › Figure 5-Plot of SUCRA for all different outcomes/Figure 5-(D) PCT.pdf]

Cumulative Probabilities

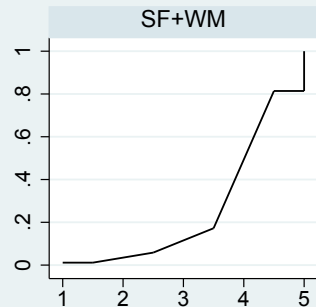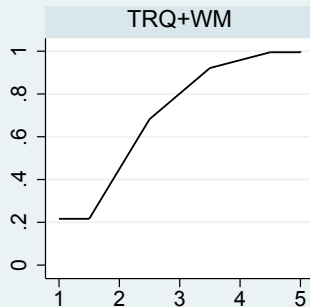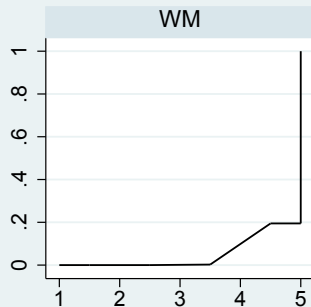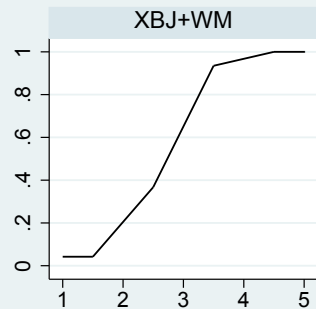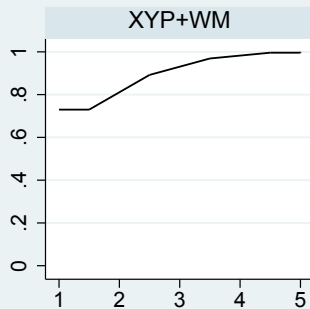

Rank

ICU length of stay

Supplement: Supplementary file 2 [file DataSheet1.ZIP › Figure 5-Plot of SUCRA for all different outcomes/Figure 5-(E) ICU length of stay.pdf]

Cumulative Probabilities

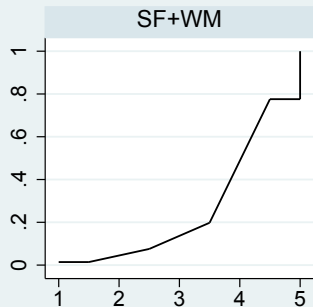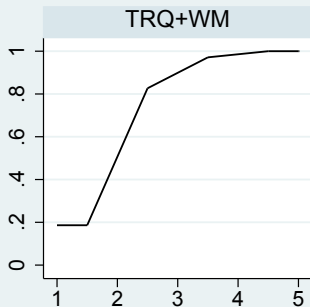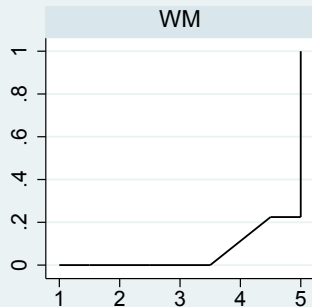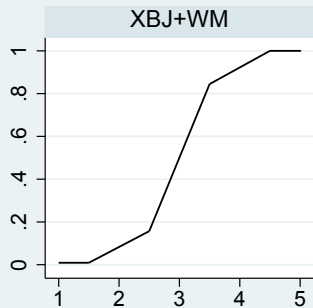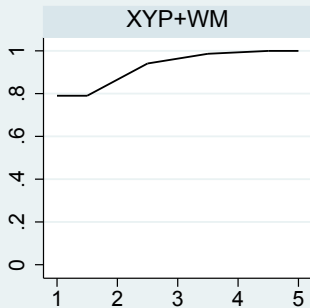

Rank

The time of mechanical ventilation

Supplement: Supplementary file 2 [file DataSheet1.ZIP › Figure 5-Plot of SUCRA for all different outcomes/Figure 5-(F) The time of mechanical ventilation.pdf]

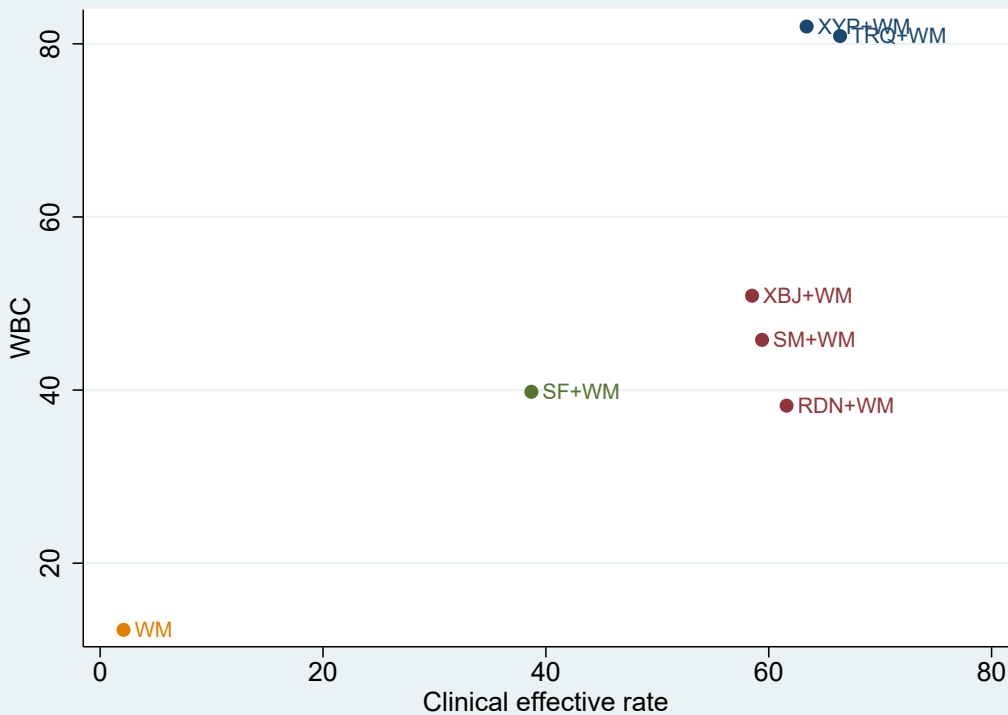

Supplement: Supplementary file 2 [file DataSheet1.ZIP › Figure 6-Cluster analysis plot for 6 outcomes/Figure 6-(A) WBC.pdf]

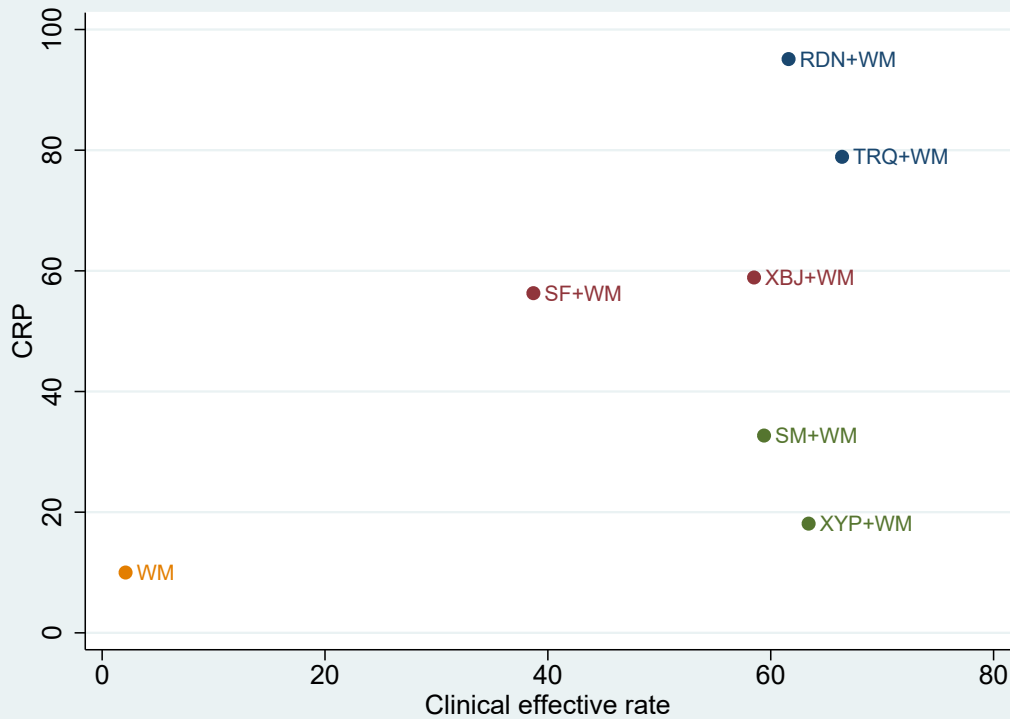

Supplement: Supplementary file 2 [file DataSheet1.ZIP › Figure 6-Cluster analysis plot for 6 outcomes/Figure 6-(B) CRP.pdf]

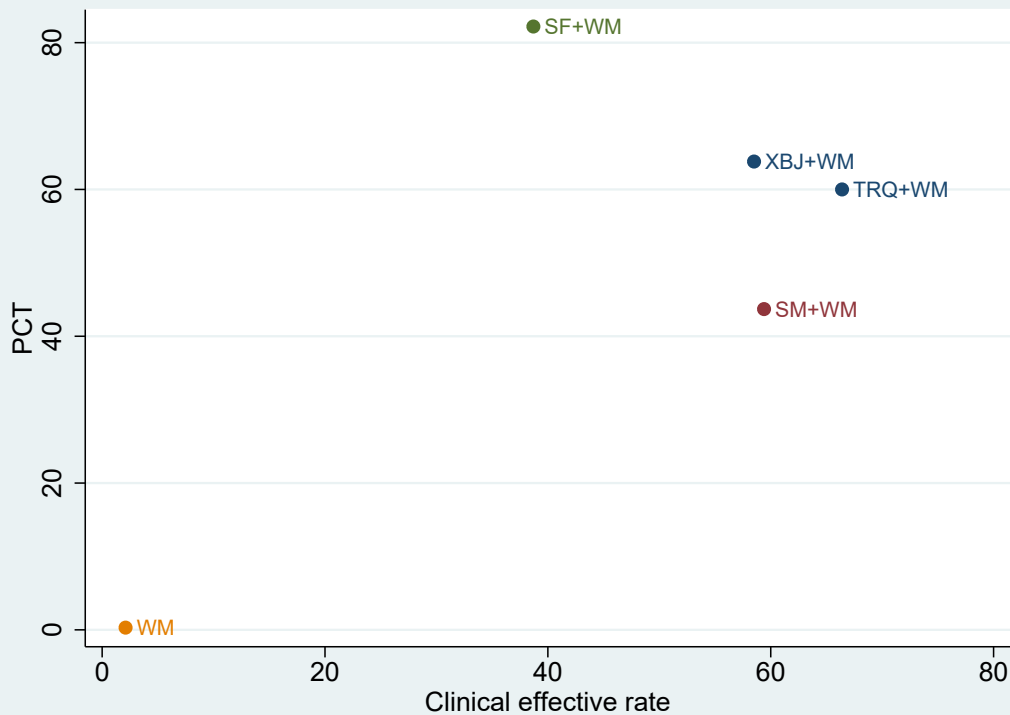

Supplement: Supplementary file 2 [file DataSheet1.ZIP › Figure 6-Cluster analysis plot for 6 outcomes/Figure 6-(C) PCT.pdf]

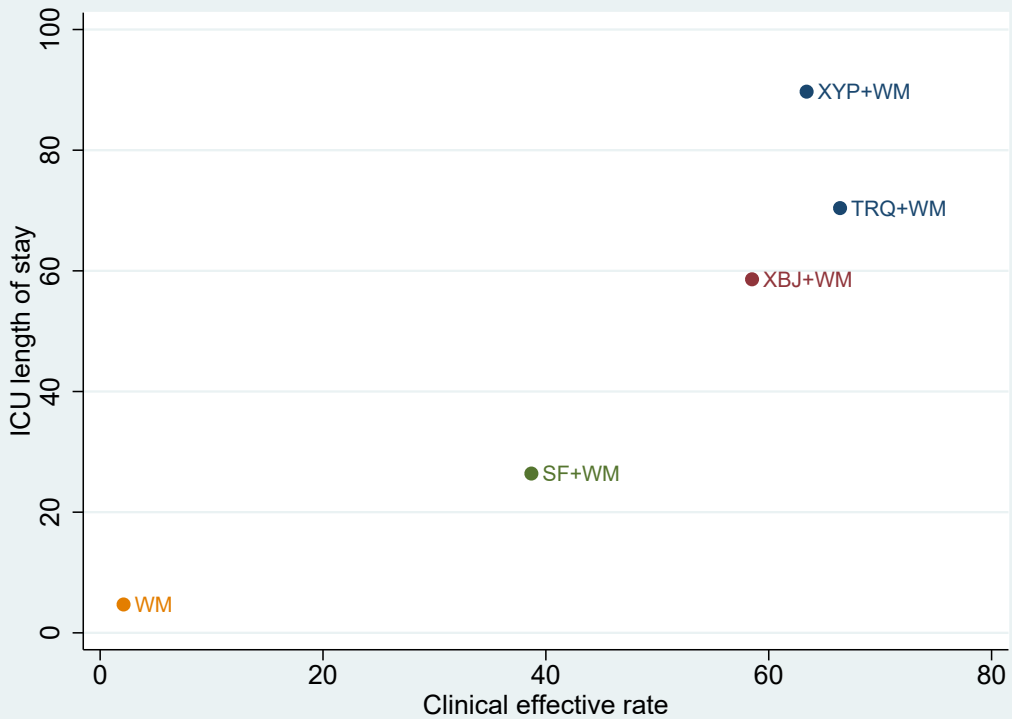

Supplement: Supplementary file 2 [file DataSheet1.ZIP › Figure 6-Cluster analysis plot for 6 outcomes/Figure 6-(D) ICU length of stay.pdf]

The time of mechanical ventilation

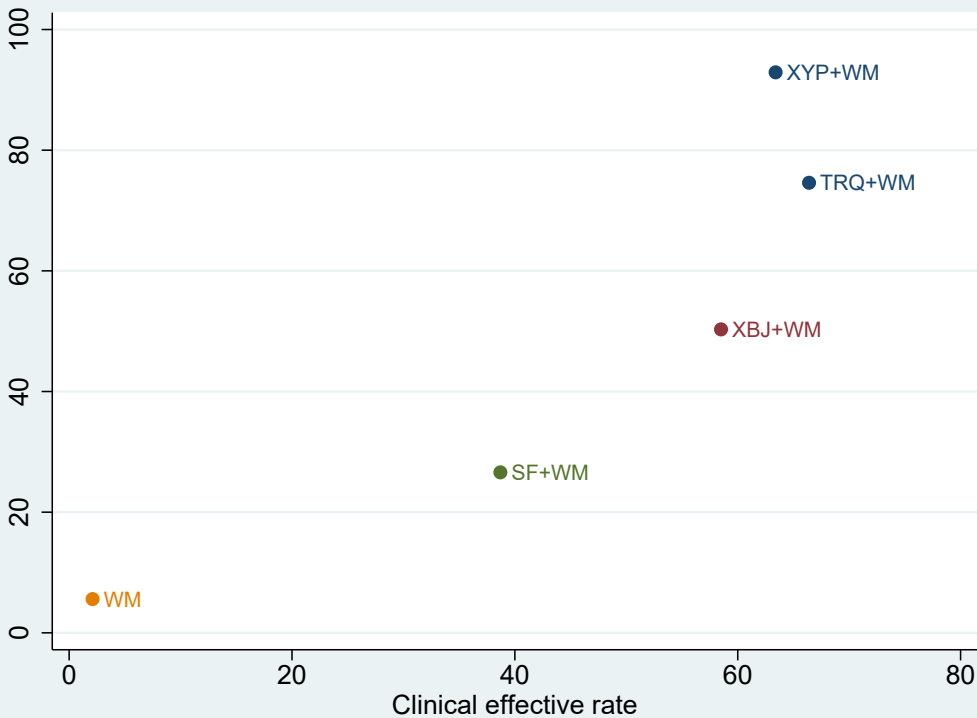

Supplement: Supplementary file 2 [file DataSheet1.ZIP › Figure 6-Cluster analysis plot for 6 outcomes/Figure 6-(E) The time of mechanical ventilation.pdf]

Standard error of logor

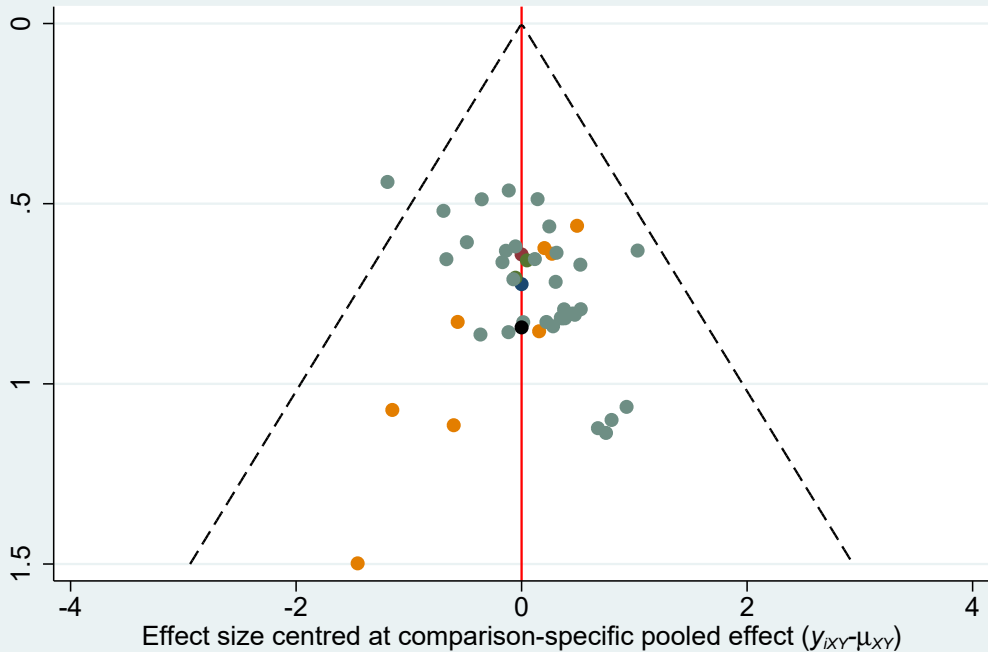

● RDN vs WM ● SF vs WM ● SM vs WM ● TRQ vs WM ● WM vs XBJ ● WM vs XYP

Supplement: Supplementary file 2 [file DataSheet1.ZIP › Figure 7-Funnel Plot/Figure 7-(A) Clinical effective rate.pdf]

Standard error of logor

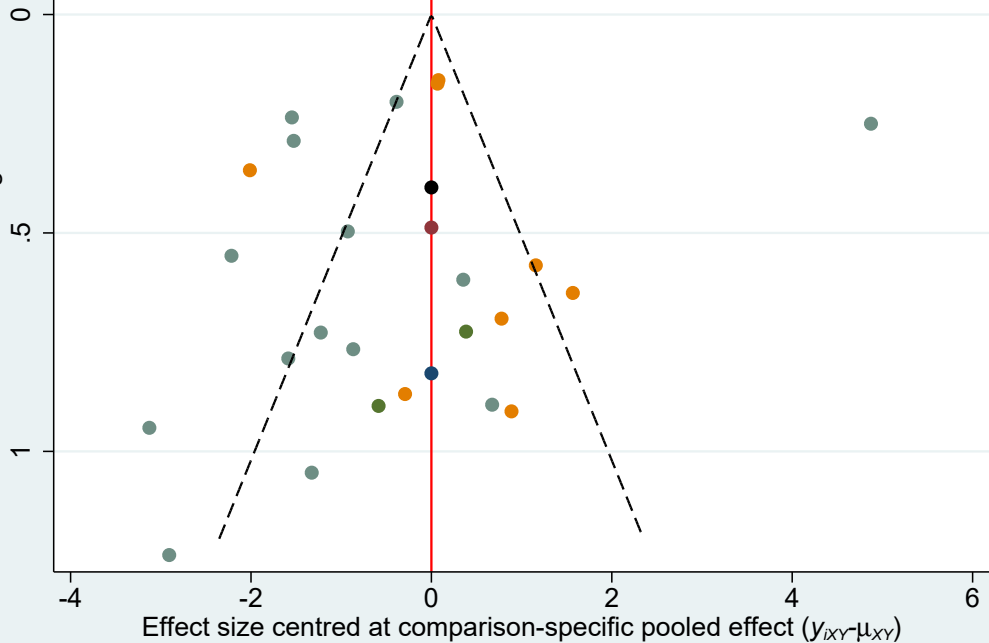

● RDN vs WM ● SF vs WM ● SM vs WM ● TRQ vs WM ● WM vs XBJ ● WM vs XYP

Supplement: Supplementary file 2 [file DataSheet1.ZIP › Figure 7-Funnel Plot/Figure 7-(B) WBC.pdf]

Standard error of logor

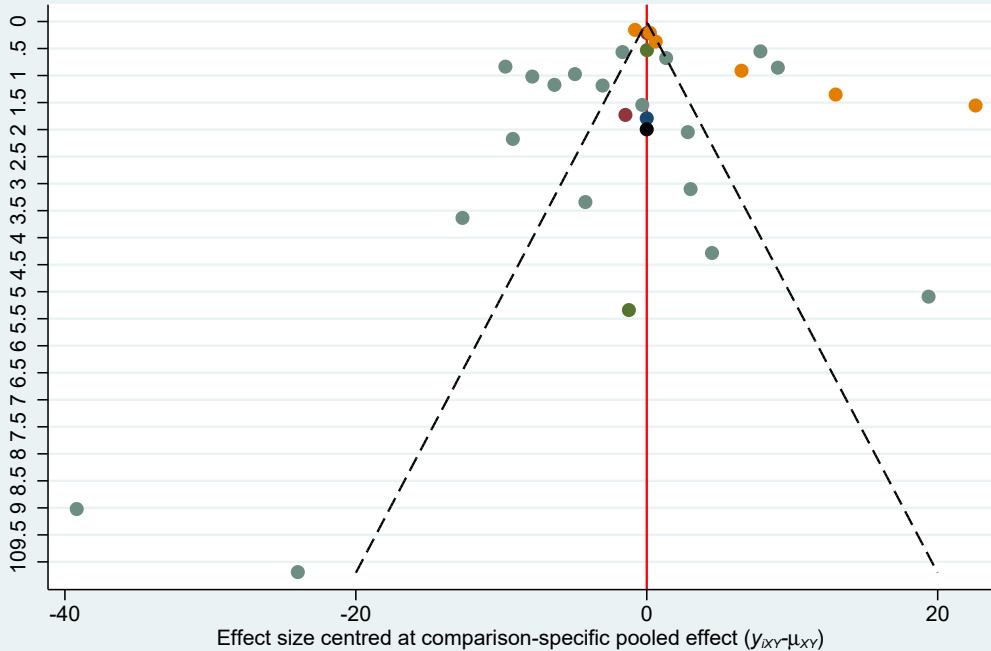

Supplement: Supplementary file 2 [file DataSheet1.ZIP › Figure 7-Funnel Plot/Figure 7-(C) CRP.pdf]

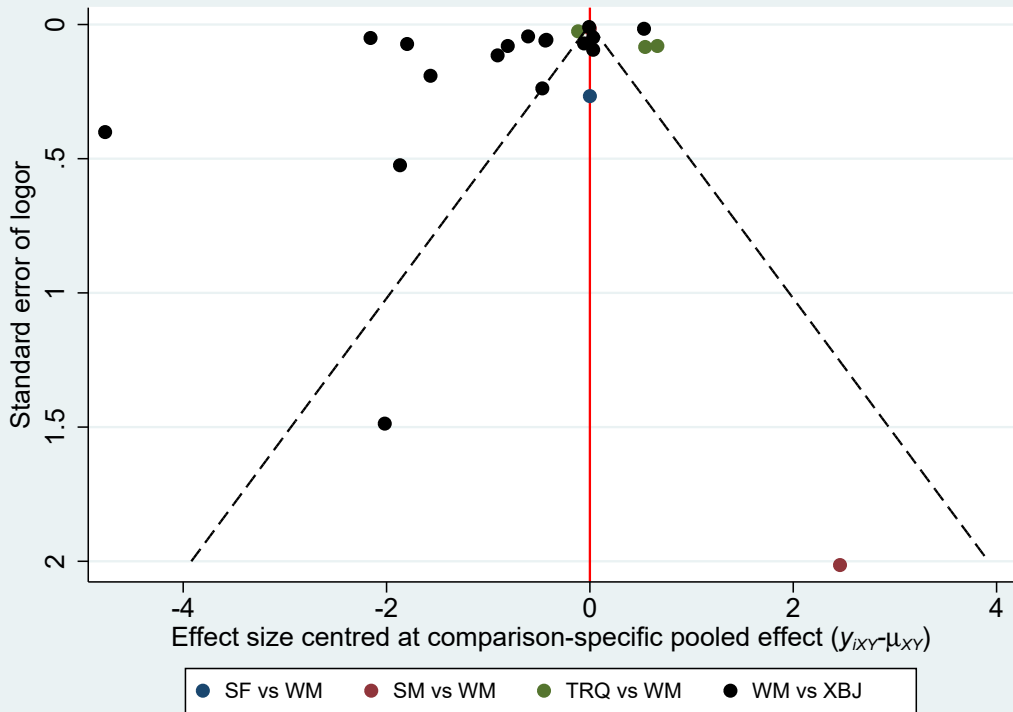

Supplement: Supplementary file 2 [file DataSheet1.ZIP › Figure 7-Funnel Plot/Figure 7-(D) PCT.pdf]

Standard error of logor

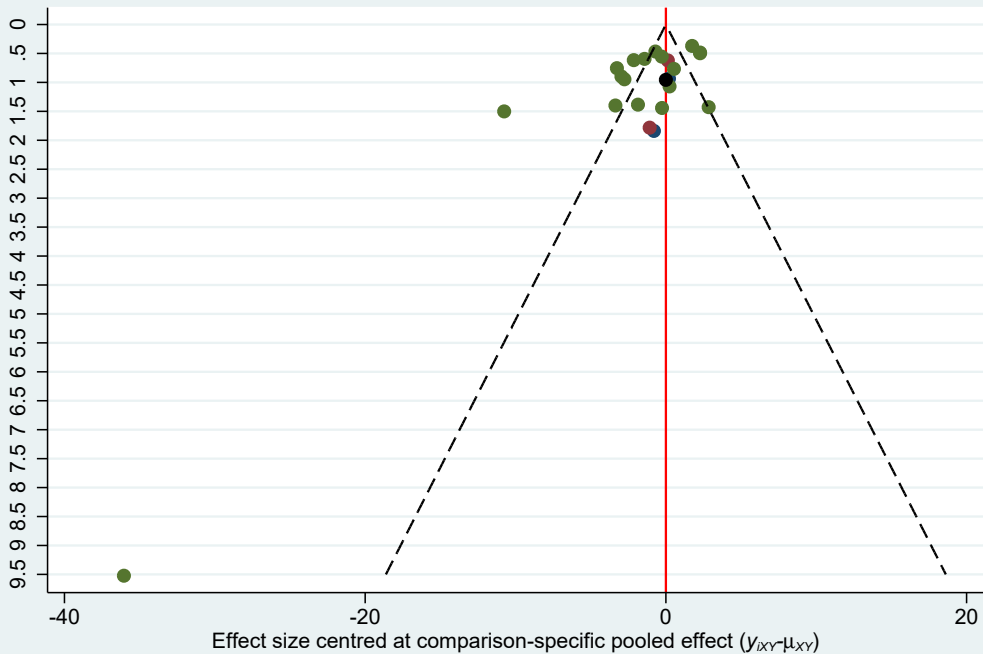

Supplement: Supplementary file 2 [file DataSheet1.ZIP › Figure 7-Funnel Plot/Figure 7-(E) ICU length of stay.pdf]

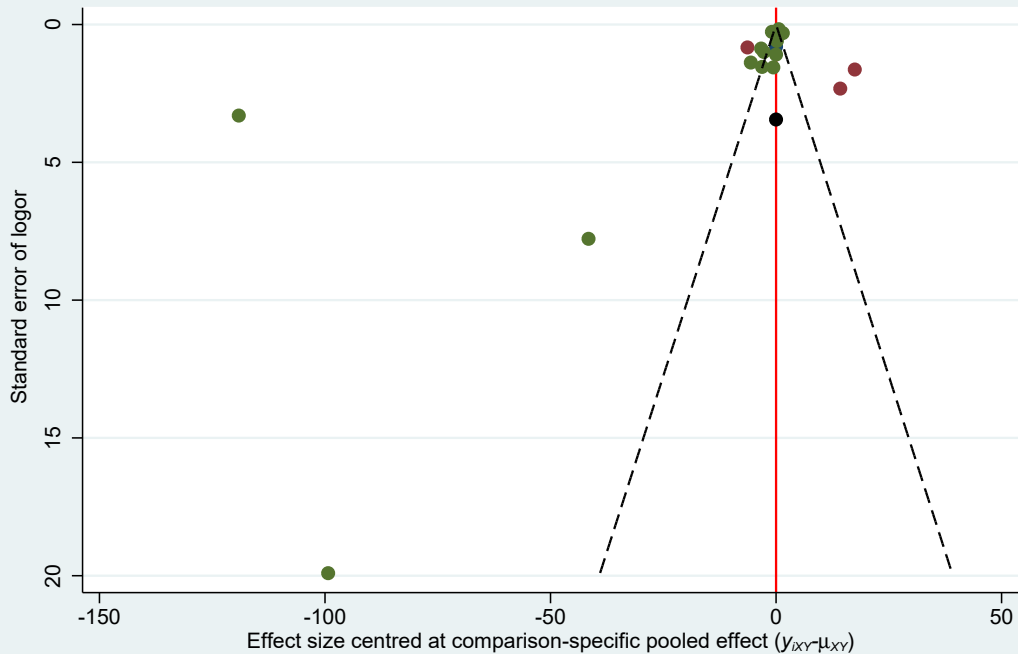

Supplement: Supplementary file 2 [file DataSheet1.ZIP › Figure 7-Funnel Plot/Figure 7-(F) The time of mechanical ventilation.pdf]
