# Supplementary material for: Comparative Efficacy of Chinese Herbal Injections for Treating Severe Pneumonia: A Systematic Review and Bayesian Network Meta-Analysis of Randomized Controlled Trials
Source: Front Pharmacol. 2022 Jan 10;12:743486. doi: 10.3389/fphar.2021.743486 (PMC8784988; doi:10.3389/fphar.2021.743486)
Supplement: Supplementary file 3 [file Table2.DOCX]

**Attachment 2: Search Strategy**

PubMed

| Number | Search terms | Results |
| --- | --- | --- |
| #1 | Severe pneumonia[MeSH Terms] | 32802 |
| #2 | Severe pneumonia[Title/Abstract] | 3560 |
| #3 | #1 OR #2 | 34356 |
| #4 | Chinese herbal injection[MeSH Terms] | 1148 |
| #5 | Chinese herbal injection[Title/Abstract] | 30 |
| #6 | Traditional Chinese medicine injection[Title/Abstract] | 83 |
| #7 | Traditional Chinese medicine[Title/Abstract] | 22500 |
| #8 | xuebijing[Title/Abstract] | 211 |
| #9 | tanreqing[Title/Abstract] | 67 |
| #10 | xiyanping[Title/Abstract] | 49 |
| #11 | reduning[Title/Abstract] | 64 |
| #12 | shenfu[Title/Abstract] | 238 |
| #13 | shenmai[Title/Abstract] | 237 |
| #14 | #4 OR #5 OR #6 OR #7 OR #8 OR #9 OR #10 OR #11 OR #12 OR #13 | 23166 |
| #15 | randomized controlled trial[Publication Type] | 527320 |
| #16 | controlled clinical trial[Publication Type] | 616568 |
| #17 | random*[All Fields] | 1448239 |
| #18 | #15 OR #16 OR #17 | 1531122 |
| #19 | #3 AND #14 AND #18 | 43 |

Web of Science

| Number | Search terms | Results |
| --- | --- | --- |
| #1 | TOPIC: Severe pneumonia | 19147 |
| #2 | TOPIC: Chinese herbal injection | 638 |
| #3 | TOPIC: Traditional Chinese medicine injection | 1641 |
| #4 | TOPIC: Traditional Chinese medicine | 27377 |
| #5 | TOPIC: xuebijing | 157 |
| #6 | TOPIC: tanreqing | 54 |
| #7 | TOPIC: xiyanping | 27 |
| #8 | TOPIC: reduning | 46 |
| #9 | TOPIC: shenfu | 267 |
| #10 | TOPIC: shenmai | 139 |
| #11 | #2 OR #3 OR #4 OR #5 OR #6 OR #7 OR #8 OR #9 OR #10 | 28172 |
| #12 | #1 AND #11 | 93 |

Cochrane Library

| Number | Search terms | Results |
| --- | --- | --- |
| #1 | severe pneumonia:ti,ab,kw | 5396 |
| #2 | Chinese herbal injection:ti,ab,kw OR Traditional Chinese medicine injection:ti,ab,kw OR Traditional Chinese medicine:ti,ab,kw OR xuebijing:ti,ab,kw OR tanreqing:ti,ab,kw | 8210 |
| #3 | xiyanping:ti,ab,kw OR reduning:ti,ab,kw OR shenfu:ti,ab,kw OR shenmai:ti,ab,kw | 360 |
| #4 | #2 OR #3 | 120 |
| #5 | randomized controlled:pt | 506157 |
| #6 | #1 AND #4 AND #5 | 2 |

Embase

| Number | Search term | Results |
| --- | --- | --- |
| #1 | 'severe pneumonia':ti,ab,kw | 4952 |
| #2 | 'chinese herbal injection':ti,ab,kw OR 'traditional chinese medicine injection':ti,ab,kw OR 'traditional chinese medicine':ti,ab,kw OR xuebijing:ti,ab,kw OR tanreqing:ti,ab,kw OR xiyanping:ti,ab,kw OR reduning:ti,ab,kw OR shenfu:ti,ab,kw OR shenmai:ti,ab,kw | 31645 |
| #3 | random:ti,ab,kw | 353333 |
| #4 | #1 AND #2 AND #3 | 2 |

CNKI

| Number | Search term | Results |
| --- | --- | --- |
| #1 | (SU='severe pneumonia' OR SU='severe pulmonary infection') AND (SU='injection' OR SU='extractive' OR SU='for injection' OR SU='Xuebijing' OR SU='Tanreqing' OR SU='Reduning' OR SU='Xiyanping' OR SU='Shenfu' OR SU='Shenmai') | 1107 |

Wanfang Data

| Number | Search term | Results |
| --- | --- | --- |
| #1 | (SU:'severe pneumonia' OR SU:'severe pulmonary infection') AND (SU:'injection' OR SU:'extractive' OR SU:'for injection' OR SU:'Xuebijing' OR SU:'Tanreqing' OR SU:'Reduning' OR SU:'Xiyanping' OR SU:'Shenfu' OR SU:'Shenmai') | 2839 |

VIP

| Number | Search term | Results |
| --- | --- | --- |
| #1 | (ti,kw='severe pneumonia' OR ti,kw='severe pulmonary infection') AND (ti,kw='injection' OR ti,kw='extractive' OR ti,kw='for injection' OR ti,kw='Xuebijing' OR ti,kw='Tanreqing' OR ti,kw='Reduning' OR ti,kw='Xiyanping' OR ti,kw='Shenfu' OR ti,kw='Shenmai') | 676 |
