# Supplementary material for: Comparative Efficacy of Chinese Herbal Injections for Treating Severe Pneumonia: A Systematic Review and Bayesian Network Meta-Analysis of Randomized Controlled Trials
Source: Front Pharmacol. 2022 Jan 10;12:743486. doi: 10.3389/fphar.2021.743486 (PMC8784988; doi:10.3389/fphar.2021.743486)
Supplement: Supplementary file 4 [file Table3.DOCX]

More details about the product information of 6 CHIs

| Injection name | Source | Species/Raw materials | Component ingredients to be measured | Botanical plant names | Therapeutic claims in TCM | Indications | Adverse drug reactions | Quality control reported? (Y/N) | Chemical analysis reporter? (Y/N) |
| --- | --- | --- | --- | --- | --- | --- | --- | --- | --- |
| XueBiJing injection | Tianjin Chasesun Pharmaceutical Co., Ltd. | *Paeoniae Radix Rubra*, A*ngelicae Sinensis Radix*, *Ligusticum Chuanxiong Hort*., Carthami Flos, *Salvia Miltiorrhiza* | Hydroxysafflor yellow A | *Paeonia lactiflora* Pall.; *Angelica sinensis* (Oliv.) Diels; *Conioselinum anthriscoides ‘Chuanxiong’*; *Carthamus tinctorius* L.; *Salvia miltiorrhiza* Bunge | Promoting blood circulation to remove blood stasis, clearing heat-toxin | Damp-heat diseases, blood-toxin stasis syndrome, such as fever, dyspnea, palpitation; systemic inflammatory response syndrome (SIRS) caused by infection; multiple organ dysfunction syndrome (MODS) | Pruritus | Y- National Drug Standard YBZ01242004-2010Z and National Pharmaceutical Standard Z20040033 issued by China Food and Drug Administration | N |
| TanReQing injection | Shanghai Kaibao Pharmaceutical Co., Ltd. | *Scutellariae Radix,*  Bear bile powder, Cornu gorais，  *Lonicerae Japonicae Flos,*  *Forsythiae Fructus* | Baicalin, >0.5mg/mL;  ursodeoxycholic Acid,﹥5.4mg/mL;  alanine, >1.75-3.005.4mg/mL | *Scutellaria baicalensis* Georgi,  Selenarctos thibetanus Cuvier,  Capra hircus Linnaeus,  *Lonicera japonica* Thunb,  *Forsythia suspensa* (Thunb.) Vahl | Clearing heat-toxin, dissipating phlegm | Phlegm heat obstruct lung syndrome, such as fever, cough, expectoration, thirst, redness of tongue, and yellow fur; acute bronchitis, acute pneumonia (early) | Dizziness, nausea, vomit, pruritus, skin rash, fever, chest congestion, edema, phlebitis, anaphylactic shock, dyspnea | Y- National Drug Standard YBZ00912003-2007Z-2009-2012 and National Pharmaceutical Standard Z20030054 issued by China Food and Drug Administration | N |
| ReDuNing injection | Jiangsu Kanion Pharmaceutical Co., Ltd. | *Artemisiae Annuae Herba,*  *Lonicerae Japonicae Flos,*  *Gardznize Fructus* | Gardenoside, 9.0-14.0mg/mL;  chlorogenic acid, 5.6-8.4mg/mL | *Artemisia annua* L.*,*  *Lonicera japonica* Thunb,  *Gardenia jasminoides* J.Ellis | Clearing heat, dispelling wind, removing toxic substance | Cold due to exogenous wind-heat, marked by high fever, headache, body pain, cough, phlegm yellow; upper respiratory tract infection and acute bronchitis | Dizziness, chest congestion, xerostomia, diarrhea, nausea, vomit, pruritus, skin rash, dyspnea | Y - National Drug Standard YBZ08202005-2009Z and National Pharmaceutical Standard Z20050217 issued by China Food and Drug Administration | N |
| XiYanPing injection | Jiangxi Qingfeng Pharmaceutical Co., Ltd. | Andrographolide sulfonate | Andrographolide sulfonate, 90.0%-110.0% of labelled amount | *Andrographis paniculata* (Burm.f.) Nees | Clearing heat-toxin, suppress cough and check dysentery | Bronchitis, tonsillitis, bacillary dysentery | Pruritus, skin rash, palpitation, diarrhea, vomit | Y - National Drug Standard WS-10863 (ZD-0863) -2002-2011Z and National Pharmaceutical Standard Z20026249 issued by China Food and National Pharmaceutical Standard Drug Administration | N |
| ShenFu injection | Ya'an Sanjiu Pharmaceutical Co., Ltd. | Red Ginseng, *Aconitum carmichaeli Debeaux* | Ginsenoside, >0.5mg/mL;  aconitine, <0.1mg/mL | *Panax ginseng* C.A.Mey.; *Aconitum carmichaeli Debeaux* | Reviving yang for resuscitation, reinforcing qi and preventing exhaustion. | Desertion syndrome caused by excessive yang-qi insufficiency (infectious, haemorrhagic and fluid loss shock); yang or qi deficiencies with palpitations, cough, stomach ache, diarrhoea, rheumatism, etc. | Pruritus, skin rash, diarrhea, nausea, vomit, anaphylactic shock | Y - WB_3_-B-3427-98-2013 issued by China Food and Drug Administration | N |
| ShenMai injection | Ya'an Sanjiu Pharmaceutical Co., Ltd.; Zhengda Qingchunbao Pharmaceutical Co., Ltd.; Hebei Shenwei Pharmaceutical Co., Ltd.; Sichuan Chuanda West China Pharmaceutical Co., Ltd.; Yunnan Gejiu Biopharmaceutical Co., Ltd.; Sichuan Shenghe Pharmaceutical Co., Ltd.; Dali Pharmaceutical Co., Ltd. | Red Ginseng, Radix Ophiopogonis | Ginsenoside, Panaxatriol, Ophiopogonone | *Panax ginseng* C.A.Mey.; *Ophiopogon japonicus* (Thunb.) Ker Gawl | Reinforcing Qi and Restoring Yang、replenishing Yin to promote production of body fluids and generating meridian | Shock, coronary heart disease, viral myocarditis, chronic pulmonary heart disease and neutropenia with qi and yin deficiencies; improves immune function of patients with tumours, combined with chemotherapy to enhance curative effects and to reduce toxic and side effects, etc. | Dizziness, diarrhea, nausea, vomit, pruritus, chest congestion, skin rash, fever, edema, phlebitis, anaphylactic shock, dyspnea, palpitation, xerostomia | Y - WS_3_-B-3428-98-2010 issued by China Food and Drug Administration | N |

Detailed information about the CHIs used in the included studies

| Study | Chinese herbal injection | Source | Species | Quality control reported? (Y/N) | Chemical analysis reported? (Y/N) |
| --- | --- | --- | --- | --- | --- |
| Qi F 2011 | XueBiJing injection | Tianjin Chasesun Pharmaceutical Co., Ltd. | *Paeonia lactiflora* Pall. [Paeoniaceae]; *Angelica sinensis* (Oliv.) Diels [Apiaceae]; *Conioselinum anthriscoides ‘Chuanxiong’* [Apiaceae]; *Carthamus tinctorius* L. [Asteraceae]; *Salvia miltiorrhiza* Bunge [Lamiaceae] | N | N |
| Li ZX 2017 | XueBiJing injection | Tianjin Chasesun Pharmaceutical Co., Ltd. | *Paeonia lactiflora* Pall. [Paeoniaceae]; *Angelica sinensis* (Oliv.) Diels [Apiaceae]; *Conioselinum anthriscoides ‘Chuanxiong’* [Apiaceae]; *Carthamus tinctorius* L. [Asteraceae]; *Salvia miltiorrhiza* Bunge [Lamiaceae] | Y - H20040033 issued by China Food and Drug Administration | N |
| Zhou SJ 2017 | XueBiJing injection | Tianjin Chasesun Pharmaceutical Co., Ltd. | *Paeonia lactiflora* Pall. [Paeoniaceae]; *Angelica sinensis* (Oliv.) Diels [Apiaceae]; *Conioselinum anthriscoides ‘Chuanxiong’* [Apiaceae]; *Carthamus tinctorius* L. [Asteraceae]; *Salvia miltiorrhiza* Bunge [Lamiaceae] | Y - Z20040033 issued by China Food and Drug Administration | N |
| Yin ZM 2019 | XueBiJing injection | Tianjin Chasesun Pharmaceutical Co., Ltd. | *Paeonia lactiflora* Pall. [Paeoniaceae]; *Angelica sinensis* (Oliv.) Diels [Apiaceae]; *Conioselinum anthriscoides ‘Chuanxiong’* [Apiaceae]; *Carthamus tinctorius* L. [Asteraceae]; *Salvia miltiorrhiza* Bunge [Lamiaceae] | Y - Z20040033 issued by China Food and Drug Administration | N |
| Shang HB 2019 | XueBiJing injection | Tianjin Chasesun Pharmaceutical Co., Ltd. | *Paeonia lactiflora* Pall. [Paeoniaceae]; *Angelica sinensis* (Oliv.) Diels [Apiaceae]; *Conioselinum anthriscoides ‘Chuanxiong’* [Apiaceae]; *Carthamus tinctorius* L. [Asteraceae]; *Salvia miltiorrhiza* Bunge [Lamiaceae] | Y - Z20040033 issued by China Food and Drug Administration | N |
| Chen S 2019 | XueBiJing injection | Tianjin Chasesun Pharmaceutical Co., Ltd. | *Paeonia lactiflora* Pall. [Paeoniaceae]; *Angelica sinensis* (Oliv.) Diels [Apiaceae]; *Conioselinum anthriscoides ‘Chuanxiong’* [Apiaceae]; *Carthamus tinctorius* L. [Asteraceae]; *Salvia miltiorrhiza* Bunge [Lamiaceae] | Y - Z20040033 issued by China Food and Drug Administration | N |
| Xin RR 2020 | XueBiJing injection | Tianjin Chasesun Pharmaceutical Co., Ltd. | *Paeonia lactiflora* Pall. [Paeoniaceae]; *Angelica sinensis* (Oliv.) Diels [Apiaceae]; *Conioselinum anthriscoides ‘Chuanxiong’* [Apiaceae]; *Carthamus tinctorius* L. [Asteraceae]; *Salvia miltiorrhiza* Bunge [Lamiaceae] | Y - China lot number: 171213 and 190523 | N |
| Xiao Q 2020 | XueBiJing injection | Tianjin Chasesun Pharmaceutical Co., Ltd. | *Paeonia lactiflora* Pall. [Paeoniaceae]; *Angelica sinensis* (Oliv.) Diels [Apiaceae]; *Conioselinum anthriscoides ‘Chuanxiong’* [Apiaceae]; *Carthamus tinctorius* L. [Asteraceae]; *Salvia miltiorrhiza* Bunge [Lamiaceae] | Y - Z20040033 issued by China Food and Drug Administration | N |
| Chen JZ 2019 | XueBiJing injection | Tianjin Chasesun Pharmaceutical Co., Ltd. | *Paeonia lactiflora* Pall. [Paeoniaceae]; *Angelica sinensis* (Oliv.) Diels [Apiaceae]; *Conioselinum anthriscoides ‘Chuanxiong’* [Apiaceae]; *Carthamus tinctorius* L. [Asteraceae]; *Salvia miltiorrhiza* Bunge [Lamiaceae] | Y - Z20040033 issued by China Food and Drug Administration and China lot number 160901 | N |
| Meng SD 2018 | XueBiJing injection | Tianjin Chasesun Pharmaceutical Co., Ltd. | *Paeonia lactiflora* Pall. [Paeoniaceae]; *Angelica sinensis* (Oliv.) Diels [Apiaceae]; *Conioselinum anthriscoides ‘Chuanxiong’* [Apiaceae]; *Carthamus tinctorius* L. [Asteraceae]; *Salvia miltiorrhiza* Bunge [Lamiaceae] | Y - China lot number: 140712 and 160801 | N |
| Wang L 2019 | XueBiJing injection | Tianjin Chasesun Pharmaceutical Co., Ltd. | *Paeonia lactiflora* Pall. [Paeoniaceae]; *Angelica sinensis* (Oliv.) Diels [Apiaceae]; *Conioselinum anthriscoides ‘Chuanxiong’* [Apiaceae]; *Carthamus tinctorius* L. [Asteraceae]; *Salvia miltiorrhiza* Bunge [Lamiaceae] | Y - China lot number: 140712 | N |
| Zhou ZY 2018 | XueBiJing injection | Tianjin Chasesun Pharmaceutical Co., Ltd. | *Paeonia lactiflora* Pall. [Paeoniaceae]; *Angelica sinensis* (Oliv.) Diels [Apiaceae]; *Conioselinum anthriscoides ‘Chuanxiong’* [Apiaceae]; *Carthamus tinctorius* L. [Asteraceae]; *Salvia miltiorrhiza* Bunge [Lamiaceae] | Y - Z20040033 issued by China Food and Drug Administration | N |
| Wang LL 2020 | XueBiJing injection | Tianjin Chasesun Pharmaceutical Co., Ltd. | *Paeonia lactiflora* Pall. [Paeoniaceae]; *Angelica sinensis* (Oliv.) Diels [Apiaceae]; *Conioselinum anthriscoides ‘Chuanxiong’* [Apiaceae]; *Carthamus tinctorius* L. [Asteraceae]; *Salvia miltiorrhiza* Bunge [Lamiaceae] | N | N |
| Wang M 2017 | XueBiJing injection | Tianjin Chasesun Pharmaceutical Co., Ltd. | *Paeonia lactiflora* Pall. [Paeoniaceae]; *Angelica sinensis* (Oliv.) Diels [Apiaceae]; *Conioselinum anthriscoides ‘Chuanxiong’* [Apiaceae]; *Carthamus tinctorius* L. [Asteraceae]; *Salvia miltiorrhiza* Bunge [Lamiaceae] | Y - China lot number: 1006121 | N |
| Tian J 2019 | XueBiJing injection | Tianjin Chasesun Pharmaceutical Co., Ltd. | *Paeonia lactiflora* Pall. [Paeoniaceae]; *Angelica sinensis* (Oliv.) Diels [Apiaceae]; *Conioselinum anthriscoides ‘Chuanxiong’* [Apiaceae]; *Carthamus tinctorius* L. [Asteraceae]; *Salvia miltiorrhiza* Bunge [Lamiaceae] | Y - Z20040033 issued by China Food and Drug Administration | N |
| Zhang SL 2014 | XueBiJing injection | Not mentioned | *Paeonia lactiflora* Pall. [Paeoniaceae]; *Angelica sinensis* (Oliv.) Diels [Apiaceae]; *Conioselinum anthriscoides ‘Chuanxiong’* [Apiaceae]; *Carthamus tinctorius* L. [Asteraceae]; *Salvia miltiorrhiza* Bunge [Lamiaceae] | N | N |
| Xie GL 2016 | XueBiJing injection | Tianjin Chasesun Pharmaceutical Co., Ltd. | *Paeonia lactiflora* Pall. [Paeoniaceae]; *Angelica sinensis* (Oliv.) Diels [Apiaceae]; *Conioselinum anthriscoides ‘Chuanxiong’* [Apiaceae]; *Carthamus tinctorius* L. [Asteraceae]; *Salvia miltiorrhiza* Bunge [Lamiaceae] | Y - Z20040033 issued by China Food and Drug Administration | N |
| Qiu JN 2021 | XueBiJing injection | Tianjin Chasesun Pharmaceutical Co., Ltd. | *Paeonia lactiflora* Pall. [Paeoniaceae]; *Angelica sinensis* (Oliv.) Diels [Apiaceae]; *Conioselinum anthriscoides ‘Chuanxiong’* [Apiaceae]; *Carthamus tinctorius* L. [Asteraceae]; *Salvia miltiorrhiza* Bunge [Lamiaceae] | Y - Z20040033 issued by China Food and Drug Administration | N |
| Xu M 2017 | XueBiJing injection | Tianjin Chasesun Pharmaceutical Co., Ltd. | *Paeonia lactiflora* Pall. [Paeoniaceae]; *Angelica sinensis* (Oliv.) Diels [Apiaceae]; *Conioselinum anthriscoides ‘Chuanxiong’* [Apiaceae]; *Carthamus tinctorius* L. [Asteraceae]; *Salvia miltiorrhiza* Bunge [Lamiaceae] | Y - Z20040033 issued by China Food and Drug Administration | N |
| Ding ZP 2020 | XueBiJing injection | Tianjin Chasesun Pharmaceutical Co., Ltd. | *Paeonia lactiflora* Pall. [Paeoniaceae]; *Angelica sinensis* (Oliv.) Diels [Apiaceae]; *Conioselinum anthriscoides ‘Chuanxiong’* [Apiaceae]; *Carthamus tinctorius* L. [Asteraceae]; *Salvia miltiorrhiza* Bunge [Lamiaceae] | Y - Z20040033 issued by China Food and Drug Administration | N |
| Sheng N 2019 | XueBiJing injection | Tianjin Chasesun Pharmaceutical Co., Ltd. | *Paeonia lactiflora* Pall. [Paeoniaceae]; *Angelica sinensis* (Oliv.) Diels [Apiaceae]; *Conioselinum anthriscoides ‘Chuanxiong’* [Apiaceae]; *Carthamus tinctorius* L. [Asteraceae]; *Salvia miltiorrhiza* Bunge [Lamiaceae] | Y - Z20040033 issued by China Food and Drug Administration | N |
| Wang DL 2019 | XueBiJing injection | Tianjin Chasesun Pharmaceutical Co., Ltd. | *Paeonia lactiflora* Pall. [Paeoniaceae]; *Angelica sinensis* (Oliv.) Diels [Apiaceae]; *Conioselinum anthriscoides ‘Chuanxiong’* [Apiaceae]; *Carthamus tinctorius* L. [Asteraceae]; *Salvia miltiorrhiza* Bunge [Lamiaceae] | Y - China lot number: 20160519 | N |
| Wang L 2018 | XueBiJing injection | Tianjin Chasesun Pharmaceutical Co., Ltd. | *Paeonia lactiflora* Pall. [Paeoniaceae]; *Angelica sinensis* (Oliv.) Diels [Apiaceae]; *Conioselinum anthriscoides ‘Chuanxiong’* [Apiaceae]; *Carthamus tinctorius* L. [Asteraceae]; *Salvia miltiorrhiza* Bunge [Lamiaceae] | Y - Z20040033 issued by China Food and Drug Administration | N |
| Wang ZW 2019 | XueBiJing injection | Tianjin Chasesun Pharmaceutical Co., Ltd. | *Paeonia lactiflora* Pall. [Paeoniaceae]; *Angelica sinensis* (Oliv.) Diels [Apiaceae]; *Conioselinum anthriscoides ‘Chuanxiong’* [Apiaceae]; *Carthamus tinctorius* L. [Asteraceae]; *Salvia miltiorrhiza* Bunge [Lamiaceae] | Y - Z20040033 issued by China Food and Drug Administration | N |
| Wei SX 2020 | XueBiJing injection | Tianjin Chasesun Pharmaceutical Co., Ltd. | *Paeonia lactiflora* Pall. [Paeoniaceae]; *Angelica sinensis* (Oliv.) Diels [Apiaceae]; *Conioselinum anthriscoides ‘Chuanxiong’* [Apiaceae]; *Carthamus tinctorius* L. [Asteraceae]; *Salvia miltiorrhiza* Bunge [Lamiaceae] | Y - Z20040033 issued by China Food and Drug Administration | N |
| Wu T 2016 | XueBiJing injection | Not mentioned | *Paeonia lactiflora* Pall. [Paeoniaceae]; *Angelica sinensis* (Oliv.) Diels [Apiaceae]; *Conioselinum anthriscoides ‘Chuanxiong’* [Apiaceae]; *Carthamus tinctorius* L. [Asteraceae]; *Salvia miltiorrhiza* Bunge [Lamiaceae] | N | N |
| Yang HW 2020 | XueBiJing injection | Tianjin Chasesun Pharmaceutical Co., Ltd. | *Paeonia lactiflora* Pall. [Paeoniaceae]; *Angelica sinensis* (Oliv.) Diels [Apiaceae]; *Conioselinum anthriscoides ‘Chuanxiong’* [Apiaceae]; *Carthamus tinctorius* L. [Asteraceae]; *Salvia miltiorrhiza* Bunge [Lamiaceae] | Y - Z20040033 issued by China Food and Drug Administration | N |
| Yang TB 2020 | XueBiJing injection | Tianjin Chasesun Pharmaceutical Co., Ltd. | *Paeonia lactiflora* Pall. [Paeoniaceae]; *Angelica sinensis* (Oliv.) Diels [Apiaceae]; *Conioselinum anthriscoides ‘Chuanxiong’* [Apiaceae]; *Carthamus tinctorius* L. [Asteraceae]; *Salvia miltiorrhiza* Bunge [Lamiaceae] | Y - Z20040033 issued by China Food and Drug Administration | N |
| Chen YJ 2014 | XueBiJing injection | Tianjin Chasesun Pharmaceutical Co., Ltd. | *Paeonia lactiflora* Pall. [Paeoniaceae]; *Angelica sinensis* (Oliv.) Diels [Apiaceae]; *Conioselinum anthriscoides ‘Chuanxiong’* [Apiaceae]; *Carthamus tinctorius* L. [Asteraceae]; *Salvia miltiorrhiza* Bunge [Lamiaceae] | N | N |
| Diao YF 2017 | XueBiJing injection | Tianjin Chasesun Pharmaceutical Co., Ltd. | *Paeonia lactiflora* Pall. [Paeoniaceae]; *Angelica sinensis* (Oliv.) Diels [Apiaceae]; *Conioselinum anthriscoides ‘Chuanxiong’* [Apiaceae]; *Carthamus tinctorius* L. [Asteraceae]; *Salvia miltiorrhiza* Bunge [Lamiaceae] | Y - China lot number: 15072101 | N |
| Zheng YN 2020 | XueBiJing injection | Tianjin Chasesun Pharmaceutical Co., Ltd. | *Paeonia lactiflora* Pall. [Paeoniaceae]; *Angelica sinensis* (Oliv.) Diels [Apiaceae]; *Conioselinum anthriscoides ‘Chuanxiong’* [Apiaceae]; *Carthamus tinctorius* L. [Asteraceae]; *Salvia miltiorrhiza* Bunge [Lamiaceae] | Y - Z20040033 issued by China Food and Drug Administration | N |
| Zhu JJ 2014 | XueBiJing injection | Not mentioned | *Paeonia lactiflora* Pall. [Paeoniaceae]; *Angelica sinensis* (Oliv.) Diels [Apiaceae]; *Conioselinum anthriscoides ‘Chuanxiong’* [Apiaceae]; *Carthamus tinctorius* L. [Asteraceae]; *Salvia miltiorrhiza* Bunge [Lamiaceae] | N | N |
| Zhuang L 2016 | XueBiJing injection | Tianjin Chasesun Pharmaceutical Co., Ltd. | *Paeonia lactiflora* Pall. [Paeoniaceae]; *Angelica sinensis* (Oliv.) Diels [Apiaceae]; *Conioselinum anthriscoides ‘Chuanxiong’* [Apiaceae]; *Carthamus tinctorius* L. [Asteraceae]; *Salvia miltiorrhiza* Bunge [Lamiaceae] | N | N |
| Zhao YJ 2019 | XueBiJing injection | Tianjin Chasesun Pharmaceutical Co., Ltd. | *Paeonia lactiflora* Pall. [Paeoniaceae]; *Angelica sinensis* (Oliv.) Diels [Apiaceae]; *Conioselinum anthriscoides ‘Chuanxiong’* [Apiaceae]; *Carthamus tinctorius* L. [Asteraceae]; *Salvia miltiorrhiza* Bunge [Lamiaceae] | Y - Z20040033 issued by China Food and Drug Administration | N |
| Pan XB 2017 | XueBiJing injection | Tianjin Chasesun Pharmaceutical Co., Ltd. | *Paeonia lactiflora* Pall. [Paeoniaceae]; *Angelica sinensis* (Oliv.) Diels [Apiaceae]; *Conioselinum anthriscoides ‘Chuanxiong’* [Apiaceae]; *Carthamus tinctorius* L. [Asteraceae]; *Salvia miltiorrhiza* Bunge [Lamiaceae] | Y - Z20040033 issued by China Food and Drug Administration | N |
| Song Y 2019 | XueBiJing injection | Tianjin Chasesun Pharmaceutical Co., Ltd. | *Paeonia lactiflora* Pall. [Paeoniaceae]; *Angelica sinensis* (Oliv.) Diels [Apiaceae]; *Conioselinum anthriscoides ‘Chuanxiong’* [Apiaceae]; *Carthamus tinctorius* L. [Asteraceae]; *Salvia miltiorrhiza* Bunge [Lamiaceae] | Y - China lot number: 1304291, 1401091, and 1501261 | N |
| Wu JH 2015 | XueBiJing injection | Tianjin Chasesun Pharmaceutical Co., Ltd. | *Paeonia lactiflora* Pall. [Paeoniaceae]; *Angelica sinensis* (Oliv.) Diels [Apiaceae]; *Conioselinum anthriscoides ‘Chuanxiong’* [Apiaceae]; *Carthamus tinctorius* L. [Asteraceae]; *Salvia miltiorrhiza* Bunge [Lamiaceae] | Y - China lot number: 1006121 | N |
| Song B 2015 | XueBiJing injection | Tianjin Chasesun Pharmaceutical Co., Ltd. | *Paeonia lactiflora* Pall. [Paeoniaceae]; *Angelica sinensis* (Oliv.) Diels [Apiaceae]; *Conioselinum anthriscoides ‘Chuanxiong’* [Apiaceae]; *Carthamus tinctorius* L. [Asteraceae]; *Salvia miltiorrhiza* Bunge [Lamiaceae] | N | N |
| Gao YQ 2014 | XueBiJing injection | Tianjin Chasesun Pharmaceutical Co., Ltd. | *Paeonia lactiflora* Pall. [Paeoniaceae]; *Angelica sinensis* (Oliv.) Diels [Apiaceae]; *Conioselinum anthriscoides ‘Chuanxiong’* [Apiaceae]; *Carthamus tinctorius* L. [Asteraceae]; *Salvia miltiorrhiza* Bunge [Lamiaceae] | N | N |
| Ma LH 2016 | XueBiJing injection | Tianjin Chasesun Pharmaceutical Co., Ltd. | *Paeonia lactiflora* Pall. [Paeoniaceae]; *Angelica sinensis* (Oliv.) Diels [Apiaceae]; *Conioselinum anthriscoides ‘Chuanxiong’* [Apiaceae]; *Carthamus tinctorius* L. [Asteraceae]; *Salvia miltiorrhiza* Bunge [Lamiaceae] | Y - Z20040033 issued by China Food and Drug Administration | N |
| Niu LL 2017 | XueBiJing injection | Tianjin Chasesun Pharmaceutical Co., Ltd. | *Paeonia lactiflora* Pall. [Paeoniaceae]; *Angelica sinensis* (Oliv.) Diels [Apiaceae]; *Conioselinum anthriscoides ‘Chuanxiong’* [Apiaceae]; *Carthamus tinctorius* L. [Asteraceae]; *Salvia miltiorrhiza* Bunge [Lamiaceae] | Y - Z20040033 issued by China Food and Drug Administration | N |
| Yuan HY 2020 | XueBiJing injection | Tianjin Chasesun Pharmaceutical Co., Ltd. | *Paeonia lactiflora* Pall. [Paeoniaceae]; *Angelica sinensis* (Oliv.) Diels [Apiaceae]; *Conioselinum anthriscoides ‘Chuanxiong’* [Apiaceae]; *Carthamus tinctorius* L. [Asteraceae]; *Salvia miltiorrhiza* Bunge [Lamiaceae] | Y - Z20040033 issued by China Food and Drug Administration | N |
| Deng Z 2021 | XueBiJing injection | Tianjin Chasesun Pharmaceutical Co., Ltd. | *Paeonia lactiflora* Pall. [Paeoniaceae]; *Angelica sinensis* (Oliv.) Diels [Apiaceae]; *Conioselinum anthriscoides ‘Chuanxiong’* [Apiaceae]; *Carthamus tinctorius* L. [Asteraceae]; *Salvia miltiorrhiza* Bunge [Lamiaceae] | Y - Z20040033 issued by China Food and Drug Administration | N |
| Han F 2012 | XueBiJing injection | Tianjin Chasesun Pharmaceutical Co., Ltd. | *Paeonia lactiflora* Pall. [Paeoniaceae]; *Angelica sinensis* (Oliv.) Diels [Apiaceae]; *Conioselinum anthriscoides ‘Chuanxiong’* [Apiaceae]; *Carthamus tinctorius* L. [Asteraceae]; *Salvia miltiorrhiza* Bunge [Lamiaceae] | N | N |
| Kong LY 2015 | XueBiJing injection | Not mentioned | *Paeonia lactiflora* Pall. [Paeoniaceae]; *Angelica sinensis* (Oliv.) Diels [Apiaceae]; *Conioselinum anthriscoides ‘Chuanxiong’* [Apiaceae]; *Carthamus tinctorius* L. [Asteraceae]; *Salvia miltiorrhiza* Bunge [Lamiaceae] | N | N |
| Zhang LL 2020 | XueBiJing injection | Tianjin Chasesun Pharmaceutical Co., Ltd. | *Paeonia lactiflora* Pall. [Paeoniaceae]; *Angelica sinensis* (Oliv.) Diels [Apiaceae]; *Conioselinum anthriscoides ‘Chuanxiong’* [Apiaceae]; *Carthamus tinctorius* L. [Asteraceae]; *Salvia miltiorrhiza* Bunge [Lamiaceae] | Y - Z20040033 issued by China Food and Drug Administration | N |
| Cai CY 2020 | TanReQing injection | Shanghai Kaibao Pharmaceutical Co., Ltd. | *Scutellaria baicalensis* Georgi [Lamiaceae],  Selenarctos thibetanus Cuvier,  Capra hircus Linnaeus,  *Lonicera japonica* Thunb [Caprifoliaceae], *Forsythia suspensa* (Thunb.) Vahl [Oleaceae] | Y - Z20030054 issued by China Food and Drug Administration | N |
| Lei JP 2017 | TanReQing injection | Shanghai Kaibao Pharmaceutical Co., Ltd. | *Scutellaria baicalensis* Georgi [Lamiaceae], Selenarctos thibetanus Cuvier, Capra hircus Linnaeus, *Lonicera japonica* Thunb [Caprifoliaceae], *Forsythia suspensa* (Thunb.) Vahl [Oleaceae] | Y - Z20030054 issued by China Food and Drug Administration | N |
| Huang SZ 2015 | TanReQing injection | Shanghai Kaibao Pharmaceutical Co., Ltd. | *Scutellaria baicalensis* Georgi [Lamiaceae], Selenarctos thibetanus Cuvier, Capra hircus Linnaeus, *Lonicera japonica* Thunb [Caprifoliaceae], *Forsythia suspensa* (Thunb.) Vahl [Oleaceae] | Y - Z20030054 issued by China Food and Drug Administration | N |
| Wu XD 2012 | TanReQing injection | Not mentioned | *Scutellaria baicalensis* Georgi [Lamiaceae], Selenarctos thibetanus Cuvier, Capra hircus Linnaeus, *Lonicera japonica* Thunb [Caprifoliaceae], *Forsythia suspensa* (Thunb.) Vahl [Oleaceae] | N | N |
| Wu H 2011 | TanReQing injection | Shanghai Kaibao Pharmaceutical Co., Ltd. | *Scutellaria baicalensis* Georgi [Lamiaceae], Selenarctos thibetanus Cuvier, Capra hircus Linnaeus, *Lonicera japonica* Thunb [Caprifoliaceae], *Forsythia suspensa* (Thunb.) Vahl [Oleaceae] | Y - Z20030054 issued by China Food and Drug Administration | N |
| Zhang HY 2021 | TanReQing injection | Shanghai Kaibao Pharmaceutical Co., Ltd. | *Scutellaria baicalensis* Georgi [Lamiaceae], Selenarctos thibetanus Cuvier, Capra hircus Linnaeus, *Lonicera japonica* Thunb [Caprifoliaceae], *Forsythia suspensa* (Thunb.) Vahl [Oleaceae] | Y - Z20030054 issued by China Food and Drug Administration | N |
| Xi R 2016 | TanReQing injection | Shanghai Kaibao Pharmaceutical Co., Ltd. | *Scutellaria baicalensis* Georgi [Lamiaceae], Selenarctos thibetanus Cuvier, Capra hircus Linnaeus, *Lonicera japonica* Thunb [Caprifoliaceae], *Forsythia suspensa* (Thunb.) Vahl [Oleaceae] | Y - Z20030054 issued by China Food and Drug Administration | N |
| Li J 2013 | TanReQing injection | Shanghai Kaibao Pharmaceutical Co., Ltd. | *Scutellaria baicalensis* Georgi [Lamiaceae], Selenarctos thibetanus Cuvier, Capra hircus Linnaeus, *Lonicera japonica* Thunb [Caprifoliaceae], *Forsythia suspensa* (Thunb.) Vahl [Oleaceae] | Y - Z20030054 issued by China Food and Drug Administration | N |
| Sun JG 2020 | TanReQing injection | Shanghai Kaibao Pharmaceutical Co., Ltd. | *Scutellaria baicalensis* Georgi [Lamiaceae], Selenarctos thibetanus Cuvier, Capra hircus Linnaeus, *Lonicera japonica* Thunb [Caprifoliaceae], *Forsythia suspensa* (Thunb.) Vahl [Oleaceae] | Y - Z20030054 issued by China Food and Drug Administration | N |
| Liu XL 2019 | TanReQing injection | Shanghai Kaibao Pharmaceutical Co., Ltd. | *Scutellaria baicalensis* Georgi [Lamiaceae], Selenarctos thibetanus Cuvier, Capra hircus Linnaeus, *Lonicera japonica* Thunb [Caprifoliaceae], *Forsythia suspensa* (Thunb.) Vahl [Oleaceae] | Y - Z20030054 issued by China Food and Drug Administration | N |
| Sun GX 2012 | ReDuNing injection | Jiangsu Kanion Pharmaceutical Co., Ltd. | *Artemisia annua* L. [Asteraceae]*,* *Lonicera japonica* Thunb [Caprifoliaceae], *Gardenia jasminoides* J.Ellis [Rubiaceae] | N | N |
| Yang ZX 2014 | XiYanPing injection | Jiangxi Qingfeng Pharmaceutical Co., Ltd. | *Andrographis paniculata* (Burm.f.) Nees [Acanthaceae] | Y - Z20026249 issued by China Food and Drug Administration | N |
| Zhang LL 2015 | XiYanPing injection | Jiangxi Qingfeng Pharmaceutical Co., Ltd. | *Andrographis paniculata* (Burm.f.) Nees [Acanthaceae] | N | N |
| Lv SJ 2017 | ShenFu injection | Shenzhen Huarun Sanjiu Pharmaceutical Trade Co. Ltd | *Panax ginseng* C.A.Mey.[Araliaceae]  *Aconitum carmichaeli Debeaux* [Ranunculaceae] | Y - Batch No.: z20043117 | N |
| Lin H 2013 | ShenFu injection | Ya'an Sanjiu Pharmaceutical Co., Ltd. | *Panax ginseng* C.A.Mey.[Araliaceae]; *Aconitum carmichaeli Debeaux* [Ranunculaceae] | N | N |
| Xia LF 2017 | ShenFu injection | Ya'an Sanjiu Pharmaceutical Co., Ltd. | *Panax ginseng* C.A.Mey.[Araliaceae]; *Aconitum carmichaeli Debeaux* [Ranunculaceae] | Y - Z51020664 issued by China Food and Drug Administration | N |
| Fan XC 2018 | ShenMai injection | Zhengda Qingchunbao Pharmaceutical Co., Ltd. | *Panax ginseng* C.A.Mey.[Araliaceae]; *Ophiopogon japonicus* (Thunb.) Ker Gawl. [Asparagaceae] | N | N |
| Yang GL 2019 | ShenMai injection | Hebei Shenwei Pharmaceutical Co., Ltd. | *Panax ginseng* C.A.Mey.[Araliaceae]; *Ophiopogon japonicus* (Thunb.) Ker Gawl. [Asparagaceae] | Y - Z1302887 issued by China Food and Drug Administration | N |
